# Matrilysin/MMP-7 Cleavage of Perlecan/HSPG2 Complexed with Semaphorin 3A Supports FAK-Mediated Stromal Invasion by Prostate Cancer Cells

**DOI:** 10.1038/s41598-018-25435-3

**Published:** 2018-05-08

**Authors:** Brian J. Grindel, Jerahme R. Martinez, Tristen V. Tellman, Daniel A. Harrington, Hamim Zafar, Luay Nakhleh, Leland W. Chung, Mary C. Farach-Carson

**Affiliations:** 1 Department of BioSciences, Rice University, Houston, TX 77005 USA; 2 Department of Diagnostic and Biomedical Sciences, University of Texas Health Science Center at Houston, School of Dentistry, Houston, TX 77054 USA; 3 Department of Computer Science, Rice University, Houston, TX 77005 USA; 4 Uro-Oncology Research Program, Samuel Oschin Comprehensive Cancer Institute at Cedars-Sinai Medical Center, Los Angeles, CA 90048 USA; 5 Present Address: Department of Cancer Systems Imaging, Division of Diagnostic Imaging, The University of Texas MD Anderson Cancer Center, Houston, TX 77030 USA; 6 Present Address: Department of Mechanical Engineering, University of Delaware, Newark, DE 19706 USA

## Abstract

Interrupting the interplay between cancer cells and extracellular matrix (ECM) is a strategy to halt tumor progression and stromal invasion. Perlecan/heparan sulfate proteoglycan 2 (HSPG2) is an extracellular proteoglycan that orchestrates tumor angiogenesis, proliferation, differentiation and invasion. Metastatic prostate cancer (PCa) cells degrade perlecan-rich tissue borders to reach bone, including the basement membrane, vasculature, reactive stromal matrix and bone marrow. Domain IV-3, perlecan’s last 7 immunoglobulin repeats, mimics native proteoglycan by promoting tumoroid formation. This is reversed by matrilysin/matrix metalloproteinase-7 (MMP-7) cleavage to favor cell dispersion and tumoroid dyscohesion. Both perlecan and Domain IV-3 induced a strong focal adhesion kinase (FAK) dephosphorylation/deactivation. MMP-7 cleavage of perlecan reversed this, with FAK in dispersed tumoroids becoming phosphorylated/activated with metastatic phenotype. We demonstrated Domain IV-3 interacts with the axon guidance protein semaphorin 3A (Sema3A) on PCa cells to deactivate pro-metastatic FAK. Sema3A antibody mimicked the Domain IV-3 clustering activity. Direct binding experiments showed Domain IV-3 binds Sema3A. Knockdown of Sema3A prevented Domain IV-3-induced tumoroid formation and Sema3A was sensitive to MMP-7 proteolysis. The perlecan-Sema3A complex abrogates FAK activity and stabilizes PCa cell interactions. MMP-7 expressing cells destroy the complex to initiate metastasis, destroy perlecan-rich borders, and favor invasion and progression to lethal bone disease.

## Introduction

Prostate cancer (PCa) remains the second most diagnosed cancer in the United States for men with approximately 26,000 deaths estimated in 2016^1^. Exploring novel mechanisms of PCa cell dispersion through the extracellular matrix (ECM) can lead to new avenues of treatment. During metastasis, PCa, and nearly all adenocarcinomas, must interact and breach multiple tissue borders rich in the large heparan sulfate proteoglycan (HSPG) ECM molecule, perlecan/HSPG2^2^. Perlecan-rich borders normally resist cell passage, and serve as tissue boundaries^2^. These borders include the glandular basement membrane^3^, the reactive stromal compartment^4^, the vasculature^5^, and bone marrow reticular matrix^2,6^, the most common site of PCa metastasis. Perlecan, through both its glycosaminoglycan (GAG) chains and protein core, binds growth factors and ECM molecules (e.g. collagen IV, laminin, and nidogen) to impact processes crucial to cancer including angiogenesis, proliferation and migration^7^. Disrupting native perlecan by proteases and GAG modifying enzymes is advantageous by not only removing the physical border perlecan stabilizes, but also potentially releasing growth factors and exposing cryptic bioactive motifs within perlecan^8^. Essentially, perlecan is a multifunctional proteoglycan that can play various roles depending on its presentation, molecular state and context.

Cleavage of perlecan can be achieved, in part, through the actions of matrix metalloproteinases (MMPs). Previously, we found perlecan in multiple forms, including when in complex with other basement membrane components, to be a ready substrate for the pro-cancer MMP, matrilysin (MMP-7)^9^. In more invasive PCa, MMP-7 is upregulated in relation to its endogenous inhibitor, tissue inhibitor of MMP 1 (TIMP-1)^10,11^, and in a murine model, overexpression of MMP-7 in PCa cells contributes to a more aggressive disease^12^. Recently, we demonstrated perlecan and MMP-7 co-localize at tissue interfaces within PCa sections, indicating sites for cleavage of perlecan exist at these tissue fronts^13^. When PCa cells encounter intact perlecan, cell-cell adhesion is favored over cell adhesion to the substratum, a clustering property that we previously mapped to the last 7 immunoglobulin (Igs) repeats in perlecan Domain IV (Domain IV-3)^9^. The tendency to form spheroids is dramatically reversed by MMP-7 cleavage of perlecan, allowing cells to disperse^9^, which mimics invasive cell activity in the tumor microenvironment. It is not known how PCa cells respond to perlecan in the native tissue environment, nor is it known how cells recognize the presence of perlecan at tissue borders.

This current study aimed to dissect PCa cell responses to intact perlecan and compare them to Domain IV-3, and to determine if enzymatic processing of perlecan by GAGases and/or MMP-7 modulates cell responses. Additionally, we used an unbiased approach to explore downstream signaling induced by PCa cell encounter with matrix perlecan. Finally, we sought to identify cell surface receptor(s) by which PCa cells interface directly with perlecan. Nearly all previous efforts have focused on integrins^14–17^; however, human perlecan lacks the canonical RGDS sequence found in the murine ortholog in domain III^16^. Additionally, our attempts to show interactions between Domain IV-3 and integrins were all unsuccessful (not shown). Exploring the literature, we noted that perlecan (trol) in *Drosophila melanogaster* enhances the semaphorin/plexin signaling axis to repulse and guide motor nerve axons to defasciculate^18^. In doing so, perlecan strongly aids in focal adhesion kinase (FAK) dephosphorylation and eventual integrin deactivation^18^. Semaphorins are best known as neuronal patterning proteins propagating repulsive/chemoattractive signals via their plexin/neuropilin receptors^19^. However, semaphorins/plexins also are critical modifiers in nearly all tissues, including, but not limited to, the immune system^20^, the cardiovascular system^21^, and bone development^22^. Given its influence in development and homeostasis, we sought to determine if any of various semaphorins and plexins in PCa cells interact with perlecan and by doing so influence cancer invasion and metastasis^23–26^.

## Materials and Methods

### Cell culture and transfection

The isogenic PCa cell lines LNCaP, C4-2, and C4-2B were cultured in 5% (v/v) heat inactivated fetal bovine serum (FBS) (Atlanta Biologicals, Lawrenceville, GA) in RPMI 1640 (Gibco, Thermo Fisher Scientific, Waltham Massachusetts) with 1 × penicillin/streptomycin (Gibco) and 1 × L-glutamine (Gibco). Cells were incubated at 37 °C in a humidified 5% (v/v) CO_2_ atmosphere and passaged at 90-95% confluency with 0.25% (w/v) trypsin-0.38% (w/v) EDTA solution (Gibco) with a 1:10 seeding density. The moderately differentiated ovarian adenocarcinoma SKOV3ip cells were cultured as described previously^27^.

For shRNA viral induction, C4-2 cells were seeded into a 12-well plate at approximately 40% confluency with normal culturing media for 24 hrs. Media was removed and replaced with culturing media containing 5 µg/mL polybrene. Either control shRNA lentivirus (sc-108080) or SEMA3A directed shRNA lentivirus (sc-36470-V) (Santa Cruz Biotechnology, Santa Cruz, CA) was added to the well and incubated in standard conditions for 24 hours after which the viral media was removed and replaced with full media for an additional 24 hrs. Transduced cells were washed with PBS, detached from the plate with trypsin-EDTA, counted and subjected to the cell-substrate activity assay as described below.

### Perlecan and recombinant protein purification and processing

Naturally occurring full length intact perlecan was purified from the conditioned media of HT-29 cells (formerly called WiDR cells) as described previously^28^. Recombinant domain I of perlecan was purified from HEK293-EBNA cells^29^, and perlecan Domain IV-3 was recombinantly produced in HEK293A cells as described previously^9^. Enzymatic processing of perlecan and recombinant fragments with heparitinase I, II, III, chondroitinase ABC and MMP-7 for adsorbing to wells was performed as before^9^.

### Cell-substrate activity assay

Assay of the effect of perlecan, recombinant perlecan domains, and antibodies as substrates were performed similarly to previous work^9^. Briefly, proteins were diluted in phosphate buffered saline (PBS), pH 7.4, at various µg/µL concentrations and allowed to adsorb to the well surface overnight in a cell incubator. After removal of the solution, wells were washed with PBS and replaced with reduced (1%) FBS culturing media containing the appropriate number of cells. For spotting protein within the well, protein substrate containing solutions in PBS were pipetted at 2 µL into the center of well and allowed to dry completely at room temperature (RT) overnight. Following a PBS wash and removal, cells were seeded in 1% FBS culturing media for the indicated times. The dispersion index was assessed with a 4 × objective microscope and quantified with the software ImageJ as described previously^9^. The higher the dispersion index the more single/dispersed cells while a lower index indicates clustered cells.

### Cell staining for F-actin

Cells were fixed with 3.7% (v/v) paraformaldehyde (Electron Microscopy Services, Hatfield, PA) in PBS for 10 minutes and washed 2 × 3 minutes with PBS. Cells were permeabilized with 0.1% Triton X-100 in PBS, followed by a PBS wash. Following blocking in 1% BSA PBS, cells were incubated with AlexaFluor phalloidin 568 (Thermo Fisher Scientific, no. A12380) (5 units/200 μl in 1% BSA PBS per well) for 20 minutes. Cells were washed with PBS three times, wherein the first wash contained DAPI. Cells were mounted in Slow Fade Gold Anti-Fade reagent (Thermo Fisher Scientific) and imaged with a Nikon TE300 inverted fluorescent microscope (Belmont, CA).

### Reverse phase protein array preparation and analysis

A reverse phase protein array (RPPA) was performed per the instructions of the University of Texas M.D. Anderson Cancer Center RPPA proteomics core. Domain IV-3 or bovine serum albumin (BSA) each was pre-treated overnight with MMP-7 (1:10, enzyme to substrate ratio) or mock as described above at RT. This mixture was coated on plates at 4 µg per well of a 12-well plate diluted in PBS overnight in a cell incubator. After removal and washing, 150,000 C4-2 cells per well were seeded onto the different substrates, incubated for 48 hours, washed with PBS, and lysates collected using the following lysis buffer: 1% (v/v) Triton X-100, 50 mM HEPES, pH 7.4, 150 mM NaCl, 1.5 mM MgCl2, 1 mM EGTA, 100 mM NaF, 10 mM Na pyrophosphate, 1 mM Na3VO4, 10% (v/v) glycerol, containing freshly added protease and phosphatase inhibitors from Roche Applied Science. After bicinchoninic acid (BCA) assay (Pierce/Thermo Fisher Scientific) to determine concentration (must be between 1–1.5 mg/mL), samples were boiled for 10 min in 4 × SDS sample buffer without any dye. Samples were stored at −80 °C until submission to core site. All samples were performed in triplicate. To create a new signaling network contrasting the clustered intact Domain IV-3 substrate state and the dispersed MMP-7 mediated state, previously used analysis methods were employed^30^. Essentially, an iterative statistical t-test between the two protein expression profiles (Domain IV-3, Domain IV-3 + MMP-7) was completed to derive edges for a novel network. Edges for the network were plotted in Cytoscape version 3.2.1 (http://cytoscape.org/).

### Western blots

Cell lysis, SDS-polyacrylamide gel electrophoresis, protein transfer and western blot were performed similarly as before^31^. Dual Color Protein Standard (Bio-Rad, Hercules, CA) was used to determine molecular weight. Antibodies used included rabbit anti-phospho-FAK (Tyr397), (Cell Signaling Technology, Danvers, MA), mouse anti-FAK clone 4.47 (EMD Millipore, Darmstadt, Germany), rabbit anti-FoxM1 (Novus Biologicals, Littleton, CO), rabbit anti-Phospho-p44/42 MAPK (ERK1/2) (Thr202/Tyr204) (D13.14.4E) (Cell Signaling Technology), rabbit anti-ERK total (Cell Signaling Technology), rabbit anti-phospho-AKT (Ser473) (D9E) (Cell Signaling Technology), mouse anti-GAPDH (EMD Millipore), rabbit anti-AKT total (Cell signaling Technology), rabbit anti p-Src (Tyr416) (Cell Signaling Technology) and mouse anti-total Src (Cell signaling Technology).

### Binding assay

Perlecan Domain IV-3 was diluted in PBS to 1 µg/mL and adsorbed to the surface of a 96-well ELISA plate overnight at 4 °C. Following removal and 1.5 hours 3% (w/v) BSA PBS blocking on a micro-well shaker, wells were incubated with either 1 µg/mL semaphorin 3 A (Sema3A)-Fc (R&D Systems, Minneapolis, MN) or non-immune IgG diluted in binding buffer (0.1% BSA, PBS, 1 mM CaCl_2_, pH 7.4) for 2 hours on a shaker at RT. In competition wells, the rabbit Sema3A antibody (Santa Cruz Biotechnology) was included at 2 µg/mL. Wells were washed three times with washing buffer (PBS, 1 mM CaCl_2_, pH 7.4) and incubated with Protein A-HRP (Thermo Scientific Fisher) to bind the IgG constant portion of Sema3A-Fc. Following washing, wells were incubated with 1-Step Ultra TMB substrate (Thermo Fisher Scientific) and stopped with 2N sulfuric acid to be read at 450 nm in a plate reader.

### Quartz crystal microbalance with dissipation (QCM-D)

QCM-D was performed on the Q-Sense Analyzer (E4) instrument (Biolin Scientific, Stockholm, Sweden) with two gold plated sensors extensively cleaned according to manufacturer’s instructions. After establishing a zero baseline with binding buffer (PBS, pH 7.4, 1 mM CaCl_2_), Domain IV-3 (10 µg/mL) was flowed over both sensors for 60 minutes at 20 µL/minute. Following binding buffer wash for 20 min at 100 µL/minute, sensors were blocked in 0.1% (w/v) BSA in binding buffer at 100 µL/minute for 30 minutes. Following another wash with binding buffer for 20 minutes at 100 µL/minute, one sensor had Sema3A-Fc (1 µg/mL for 20 minutes) in BSA binding buffer flowed. The other control sensor only has BSA binding buffer flowing at same rate. After allowing binding, binding buffer was flowed over for an additional 20 minutes at 100 µL/minute to observe any reversal of binding. The raw resonance frequency F3 (*Hz*) data is reported, but every available resonance and dissipation value was recorded simultaneously.

### *In silico* proteolytic analyses and statistics

To predict MMP-7 mediated cleavage sites within Sema3A, the free online software at Site Prediction was utilized (http://www.dmbr.ugent.be/prx/bioit2-public/SitePrediction/)^32^. A two tailed Student’s T-test was used to determine differences between groups in western blot, cell clustering activity, and cell binding assays. A p-value <0.05 was considered significant.

## Results

### Perlecan induced clustering is reversed by MMP-7 activity

**Figure 1:**
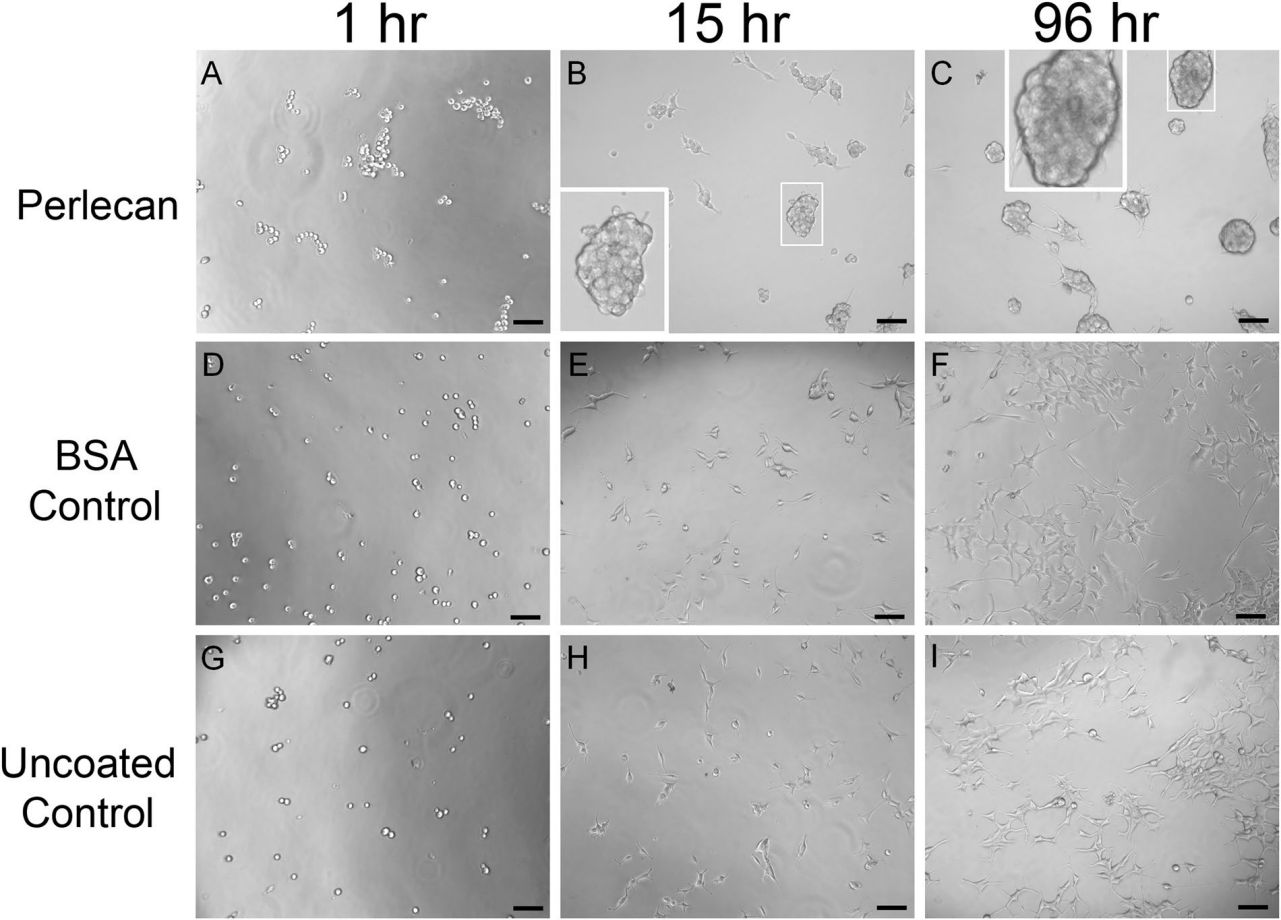
Full length perlecan induces clustering in prostate cancer cells over time. C4-2B cells were seeded into wells coated with perlecan (**A–C**), BSA (**D–F**), or tissue culture plate only (**G–I**) and imaged over a 96-hour period. Cells on perlecan begin clustering at one hour and is more pronounced later (15 and 96 hours) (white boxed inset enlarges clusters). Cells seeded on BSA coated plates lack any observable difference between the plate only, becoming adherent and spreading out in both cases. Scale bars: 100 µm.

We previously found that the most C-terminal region of perlecan domain IV, Domain IV-3, induced PCa cells to cluster when encountered as a substrate^9^. To determine if this also was a property of intact perlecan as seen by PCa cells, heparan/chondroitin sulfate containing full length human perlecan was purified from natural sources (HT29 cell conditioned media)^28^ and coated onto cell culture plates. The PCa bone metastatic prostate cancer cell line, C4-2B, was seeded on top of the substrate coated with perlecan and monitored by phase contrast microscopy over time as seen in Fig. 1, panels A-C. The cells plated as a single cell suspension onto a control non-adhesive protein substrate, bovine serum albumin (BSA) (Fig. 1D-F) and those plated onto an uncoated tissue culture plate (Fig. 1G–I) eventually attached and spread to form a monolayer, as expected. In contrast, cells plated in a single cell suspension onto the coated perlecan substrate immediately clustered within the first hour after plating. The clusters were pronounced by 15 hours, and become progressively larger over time (shown at 96 hours, panel 1 C). Boxed insets provide greater detail of the grape-like cell clusters that formed, indicating that the presence of immobilized intact perlecan favored cell-cell versus cell-substratum adhesion. While not all cell lines tested responded in this way to perlecan, this clustering effect of intact perlecan was not limited to only C4-2B PCa cells. Several cell lines including LNCaP, C4-2, the ovarian cancer cell line, SKOV3ip, and, to a limited degree, PC-3 cells also clustered on perlecan coated substrata (not shown).

**Figure 2:**
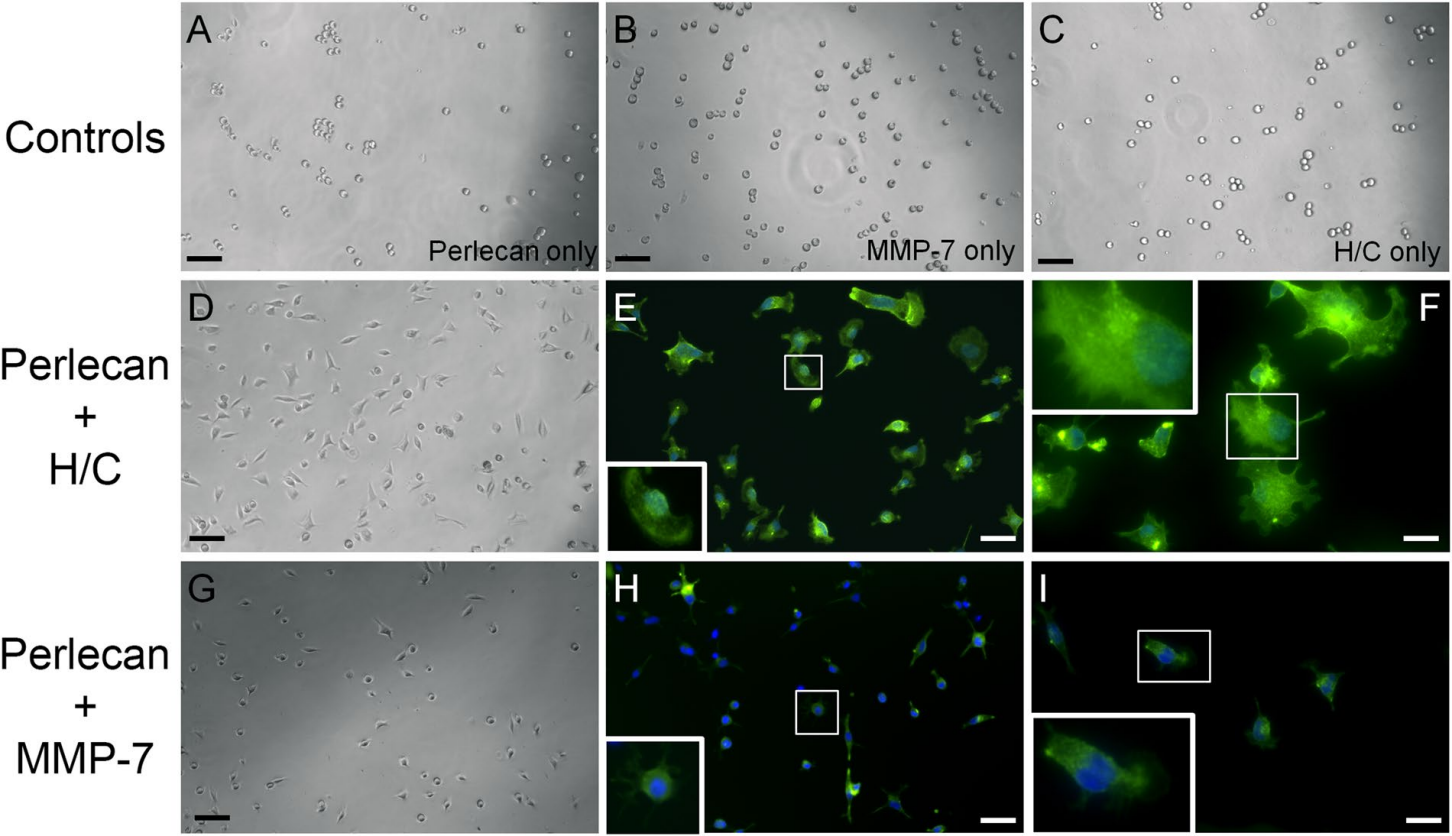
Heparanase/chondroitinase and MMP-7 enzymatic processing of perlecan favors cell substratum versus cell-cell adhesion. C4-2B cells were seeded onto various types of perlecan and control samples pre-adsorbed to the plate and imaged by bright field microscopy and then stained for F-actin (green) and nuclei (blue) after 1 hour. Perlecan digested with a mixture of heparitinase and chondroitinase ABC (H/C) (panels D–F) or MMP-7 (panels G–I) adhered to the plates within one hour and remained attached through the staining procedure (**E**,**F** and **H**,**I**). Neither intact perlecan (**A**), MMP-7 alone (**B**), nor H/C mixture alone (**C**) supported this rapid adherence to substratum, and cells remained round or formed loose clusters. Boxed insets provide an enlarged image to show cell morphology. Scalebars: 100 µm for A-D and G; 50 µm for E and H; 25 µm for F and I.

As mentioned above, HSPGs such as perlecan can be degraded by enzymes, both proteases and glycosaminoglycanases, which are secreted into the ECM during cancer progression. Heparanases, chondroitinases, and MMPs can release bioactive pieces of the intact molecule, allowing the release of bound growth factors and exposing cryptic sites in the core protein. To demonstrate this, perlecan was enzymatically digested with a mixture of heparitinases and chondroitinases (H/C) to remove the large GAG chains. Moreover, perlecan was incubated with MMP-7/matrilysin to create proteolytic fragments and to determine if MMP-7 fragmentation was sufficient to destroy the active moieties in full length perlecan that were responsible for PCa cell clustering. All digestion product mixtures and appropriate controls were coated onto cell culture wells, and subsequently C4-2B cells were plated into these coated wells. The results are shown in Fig. 2, where it is readily seen that the enzymatic digestion of perlecan led to a dramatically different cell behavior than seen in cells plated onto full length perlecan (Fig. 2A) or onto enzyme-only controls (panels B and C). Degrading the core protein either by removing the GAG chains with H/C (Fig. 2D–F), or by digesting the core protein with MMP-7 (G-I) resulted in rapid (within one hour) C4-2B cell adherence to the digested perlecan substrata, and eliminated clustering. Fixing and staining of cells that had attached after one hour to detect F-actin (green) or nuclei (blue) revealed evidence of fanning edge morphology and outreaching lamellipodia (panels E, F, H and I). Given the observation that the GAG digestion of perlecan completely removed clustering activity, we considered that the GAG chains alone are responsible for the clustering effect. However, domain I with intact HS/CS chains as a substrate does not trigger clustering akin to full length perlecan (data not shown). This reiterates that the protein core is responsible for the effect. Overall, these findings demonstrate that intact perlecan’s cell clustering effect is completely reversed upon enzymatic digestion of GAG chains or upon proteolysis by MMP-7 to favor loss of cell-cell adhesion, increase in cell substratum adhesion and ultimately, favor tumor dyscohesion and dispersion. Further supporting this, we found that another bone metastatic but osteolytic prostate cancer cell line, PC3, did not cluster nearly as well as C4-2B cells when plated onto Domain IV-3 substrata (not shown). Interestingly, this cell line expresses high levels of MMP-7^33^, which we suggest inactivates the Domain IV-3 prior to its ability to initiate a clustering effect.

### Perlecan Domain IV-3 acts as a strong border molecule that affects multiple signaling pathways

**Figure 3:**
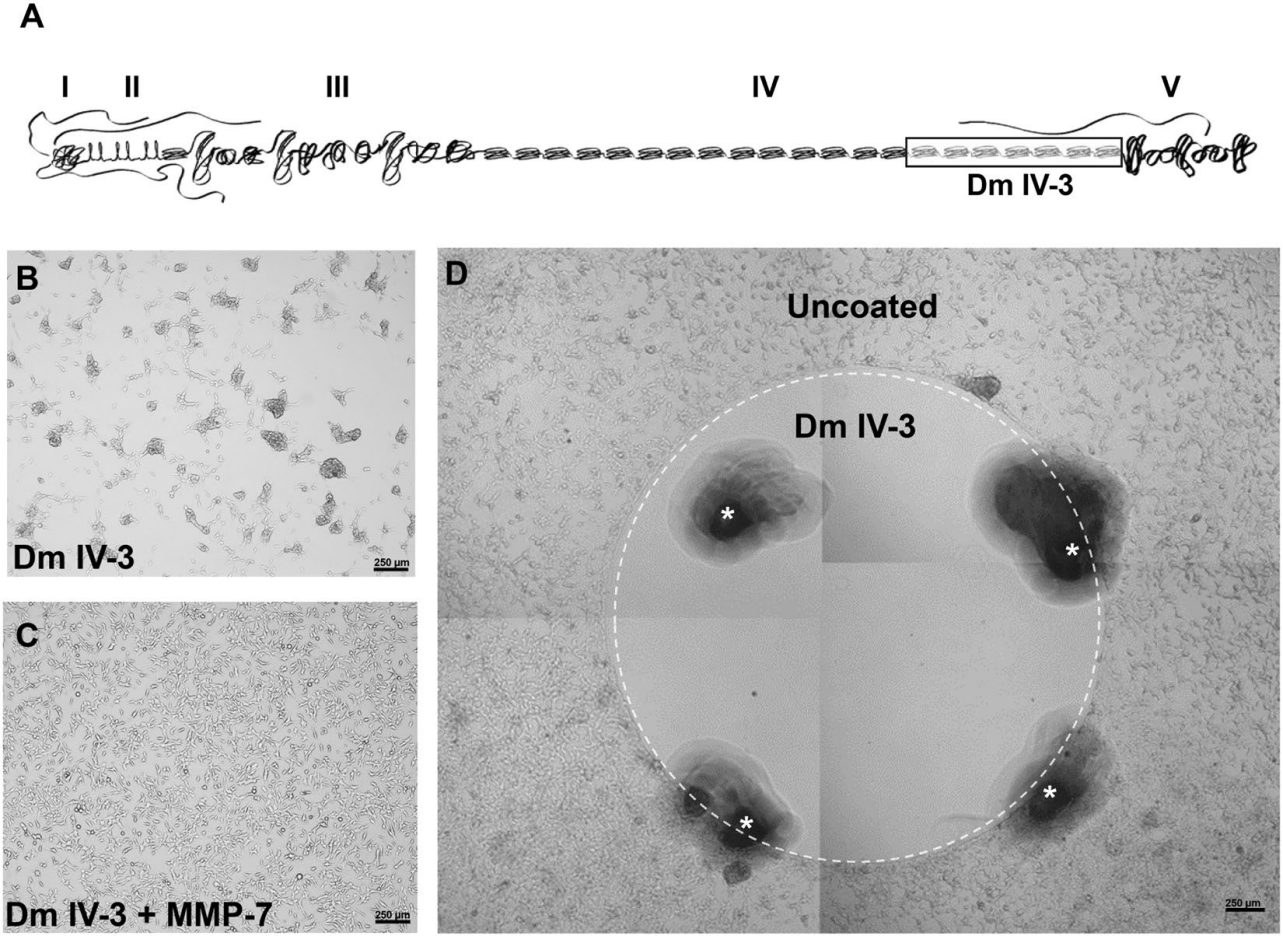
Perlecan Domain IV-3 mediates a strong repulsive/clustering effect on C4-2 cells as does full length perlecan. (**A**) Schematic of full length perlecan highlighting Domain IV-3 (the last 7 Ig repeats of Domain IV). A reverse phase protein array (RPPA) was performed on cells cultured on wells entirely coated with Domain IV-3 (panel B) or MMP-7 cleaved Domain IV-3 (panel C) for 48 hours. Cells cluster on Domain IV-3 and this is reversed by pre-cleavage with MMP-7. D.) C4-2 cells 72 hours after being cultured on a spot (inside the white dashed circle) of surface adsorbed Domain IV-3. Cells cleared out of the area, creating a border around Domain IV-3. Scalebars are 250 µm. White asterisks indicate fiduciary markers (black areas) used to approximate the location of the spotted Domain IV-3 at the time.

Previously published work from our lab established that the last 7 Ig modules contained in domain IV of perlecan (Domain IV-3) can alone account for the clustering effect that is observed for multiple PCa cell lines plated onto coated substrata^9^, but no mechanism was determined. Figure 3A provides a schematic of perlecan outlining the five domains and highlights the active region, Domain IV-3. To gain a mechanistic understanding of the downstream signaling mechanisms induced during clustering on perlecan/Domain IV-3 substrata, a reverse phase protein array (RPPA) was utilized initially as a broad unbiased screen. C4-2 cells were cultured on either Domain IV-3 or MMP-7 pre-cleaved Domain IV-3 substrata for 48 hours, lysed and prepared for the RPPA assay (controls coated with BSA +/-MMP-7 also were performed in tandem). As shown in Fig. 3, Domain IV-3 induced PCa cell clustering that persisted for 48 hours (panel B) but was completely reversed by MMP-7 proteolysis (panel C). This effect duplicates that of full length perlecan shown in Fig. 1 and re-iterates earlier findings^9^. Cells in clustered or non-clustered dispersed states were analyzed by RPPA through iterative protein-pair statistical methods developed previously^30^. This statistical method compares the pathways that are altered in the transition between the two states: clustered on Domain IV-3 and dispersed on MMP-7 cleaved Domain IV-3. From the results, we developed a *de novo* protein interaction network (supplemental Figure S1). Notably, we observed significant changes in phosphorylation status both of the FAK and Src, which pointed to a cell-substratum binding alteration that could account for a clustered versus dispersed state. Refusal of cells to attach to the domain IV-3 adsorbed surface was clearly seen by live imaging of cells seeded onto a substratum onto which a circular spot of perlecan domain IV-3 had been coated (supplemental video 1, Fig. 3D). Within 10 minutes, C4-2 cells seeded within the circle of Domain IV-3 quickly adhered to one another in active avoidance of the substrate. Cells outside the coated area have not yet attached, but do not cluster as on Domain IV-3. After 72 hours, cells formed a bordered ring around Domain IV-3 (Fig. 3D) dramatically demonstrating the high repulsive effect of this region of the perlecan core protein.

### Contact with Domain IV-3 rapidly dephosphorylates focal adhesion kinase and alters downstream signaling components

**Figure 4:**
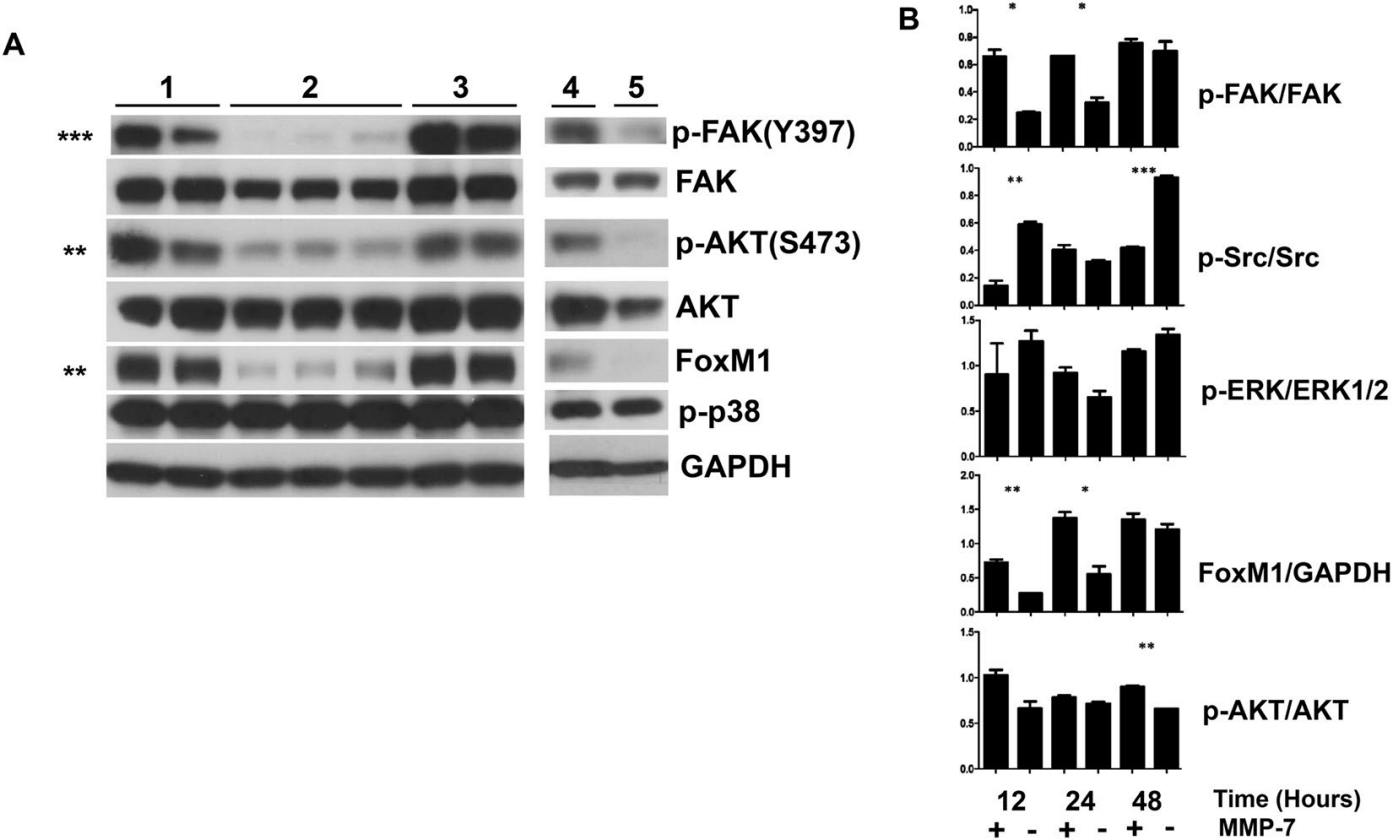
Full length perlecan and Domain IV-3 strongly dephosphorylate focal adhesion kinase (FAK) and alter several downstream signaling components. (**A**) C4-2 cells were cultured on several substrates (Set 1: Control BSA/digest buffer, set 2: Domain IV-3 alone, set 3: Domain IV-3 + MMP-7, set 4: perlecan + MMP-7, and set 5: perlecan alone) for 24 hours and probed for phosphorylated FAK (p-FAK), total FAK, p-AKT, FoxM1, p-p38, and GAPDH. Unpaired student t-tests between sets 2 and 3 were performed for densitometry quantified values of p-FAK/FAK, p-AKT/AKT, FoxM1/GAPDH, and p-p38/GAPDH. (**B**) A 12, 24 and 48-hour time course of C4-2 cells cultured on Domain IV-3 incubated either alone (−) or with MMP-7 (+) and probed for signaling components that were quantified by densitometry. An unpaired student’s t-test measured any significant difference between cells on Domain IV-3 or Domain IV-3 pre-cleaved with MMP-7 at each time point. p-values: * < 0.05, ** < 0.01, *** < 0.001. Expanded view of blots included in composite figure included in supplemental data file.

To validate important RPPA findings, western blots were performed using C4-2 cell lysates from cells that had been cultured on plates coated on various substrata for different times (Fig. 4). C4-2 cells were cultured for 24 hours on control buffer/BSA (Fig. 1A, set 1), Domain IV-3 (set 2), Domain IV-3 digested by MMP-7 (set 3), full length perlecan digested with MMP-7 (set 4), or full length perlecan (set 5). As informed by the RPPA, p-FAK (Tyr397) was highly suppressed by both Domain IV-3 and full length perlecan. As well, p-AKT (Ser473) was modestly suppressed by Domain IV-3 and perlecan. However, no change was observed in p-p38 at 24 hours. Interestingly, a novel protein interaction between p-FAK and the transcription factor FoxM1 was observed in the RPPA generated protein interaction network. As seen by immunoblotting, FoxM1 followed a similar pattern as seen with p-FAK and p-AKT, and was suppressed when cultured on Domain IV-3 and perlecan. To better understand signaling dynamic differences between the clustered (Domain IV-3) and dispersed (Domain IV-3 + MMP-7), a time course over 48 hours was performed (Fig. 4B). At 12, 24, and 48-hour time points, C4-2 cell lysates were collected after culture on Domain IV-3 or MMP-7 pre-cleaved Domain IV-3, then blotted to detect levels of p-FAK, p-Src, p-ERK, FoxM1, and p-AKT along with total protein counterparts and a GAPDH loading control. MMP-7 pre-cleavage of Domain IV-3 negated both clustering and downstream signaling triggered by Domain IV-3, as shown in Fig. 4A,B. At the earlier time points (12 and 24 hours), p-FAK was suppressed by Domain IV-3, but returned to levels similar to cleaved Domain IV-3 by 48 hours. MMP-7 cleaved Domain IV-3 didn’t change the p-FAK levels over the 48 hour period. Unexpectedly, Src phosphorylation (p-Tyr416) showed a dramatic cycle being highly phosphorylated in C4-2 cells in comparison to a substrate pre-cleaved by MMP-7 at 12 and 48 hours by Domain IV-3, but unchanged at 24 hrs. p-Src arose as a central node in the RPPA which is reflective of the 48-hour time point up-regulation in comparison to cleaved Domain IV-3 in the array. ERK phosphorylation was unchanged between intact and MMP-7 cleaved Domain IV-3 except for a non-significant downregulation at 24 hours. FoxM1 downregulation by Domain IV-3 in comparison to MMP-7 cleaved IV-3 was apparent at 12 hours, but the difference was lost at 48 hours. Modest AKT phospho-suppression by Domain IV-3 was maintained throughout the time course, but only significant at 48 hours. Overall, these experiments point to an early and dramatic decrease in p-FAK on Domain IV-3/perlecan that is completely reversed by MMP-7 activity, resulting in modulation of key downstream signaling components that would affect cell-cell versus cell-substratum adhesion.

### Semaphorin 3A is an interacting partner mediating perlecan cluster activity

**Figure 5:**
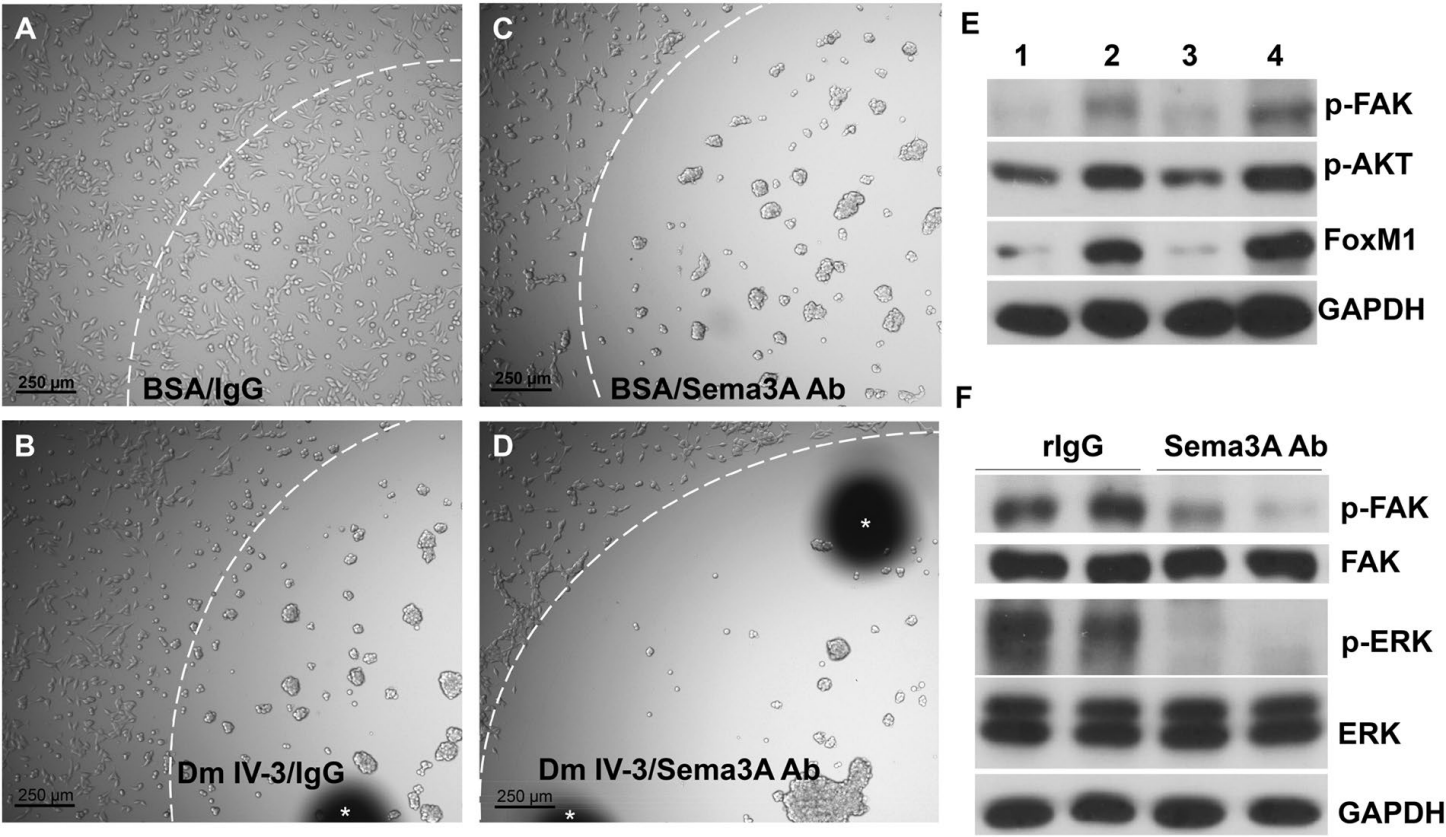
Semaphorin 3A (Sema3A) antibody imitates Domain IV-3 activity on prostate cancer cells. Cell culture wells were spotted with control BSA and rabbit IgG (panel A), Domain IV-3 and rabbit IgG (panel B), BSA and Sema3A antibody (Ab) (panel C), or Domain IV-3 and Sema3A antibody (panel D) (inside the white dashed arc line). C4-2 cells were seeded and imaged 24 hours later. Scalebar is 250 µm. White asterisks indicate black fiduciary markers used to approximate the area of surface adsorbed substrate. (**B**) A western blot of C4-2 cells cultured for 24 hours on Domain IV-3 (lane 1), rabbit IgG (lane 2), Sema3A antibody (lane 3), or control BSA (lane 4). (**C**) Western blot of C4-2 cells cultured for 24 hours on control rabbit IgG or Sema3A antibody and probed for p-FAK, FAK, p-ERK, ERK1/2, and GAPDH. The Sema3A antibody as a substrate produces results akin to Domain IV-3 for C4-2 cells.

As noted in the introduction, the strong suppression of p-FAK and the repulsive activity of perlecan are highly reminiscent of a genetic interaction between perlecan (trol) and semaphorin previously seen in *Drosophila melanogaster*. The perlecan core in conjunction with invertebrate Sema1A greatly suppressed p-FAK and integrin activity leading to axon repulsion^18^. Sema3A is a well-known axon repulsive/guidance molecule in vertebrates^34^, and is known to cause deadhesion with lack of motility in PCa cells^35^ along with its co-receptors neuropilin-1 and plexin-A1^36^. We hypothesized that in human PCa cells, Sema3A could be involved in directing perlecan’s clustering/repulsive effect. Initial experiments using a rabbit C-terminal binding Sema3A antibody provided evidence that perlecan Domain IV-3 and Sema3A interact (Fig. 5). Domain IV-3, Sema3A antibody, rabbit IgG (control to Sema3A antibody), and BSA (control to Domain IV-3) each were spotted as a substrate in various combinations on cell culture plates to observe any effect on C4-2 cell behavior. All surfaces within the white dashed arc (upper left of the circle) are coated with the indicated substrate, while everything outside is uncoated cell culture plate. Figure 5A demonstrates that after 24 hours C4-2 cells have adhered to plates coated with control BSA/IgG. Domain IV-3/IgG coating triggers the expected clustering result (Fig. 5B). Surprisingly, the C-terminal directed Sema3A antibody (Sema3A Ab in Fig. 5C) mimicked the effect of Domain IV-3, leading to C4-2 clustering. Combining Domain IV-3 and the Sema3A antibody results in an exaggerated repulsion/clustering effect (Fig. 5D), further supporting the notion that Domain IV-3 and the antibody engage the same pathway. Other specific antibodies lacked a clustering or blocking effect including anti-neuropilin 1 and a separate Sema3A antibody that does not bind the C-terminal region of Sema3A (not shown). Figure 5E,F show that molecular signatures are similar between Domain IV-3 and the Sema3A antibody as well. Cells exposed to a Domain IV-3 and Sema3A antibody substrate (Fig. 5E, lanes 1 and 3) for 24 hours have nearly identical suppression patterns for p-FAK, p-AKT, and FoxM1 in comparison to control IgG and BSA (lanes 2 and 4, respectively). Furthermore, surface adsorbed Sema3A antibody lowered ERK1/2 phosphorylation concomitantly with induction of strong FAK dephosphorylation in comparison to non-immune rabbit IgG controls (Fig. 5F).

### Sema3A interacts with Perlecan Domain IV-3

**Figure 6:**
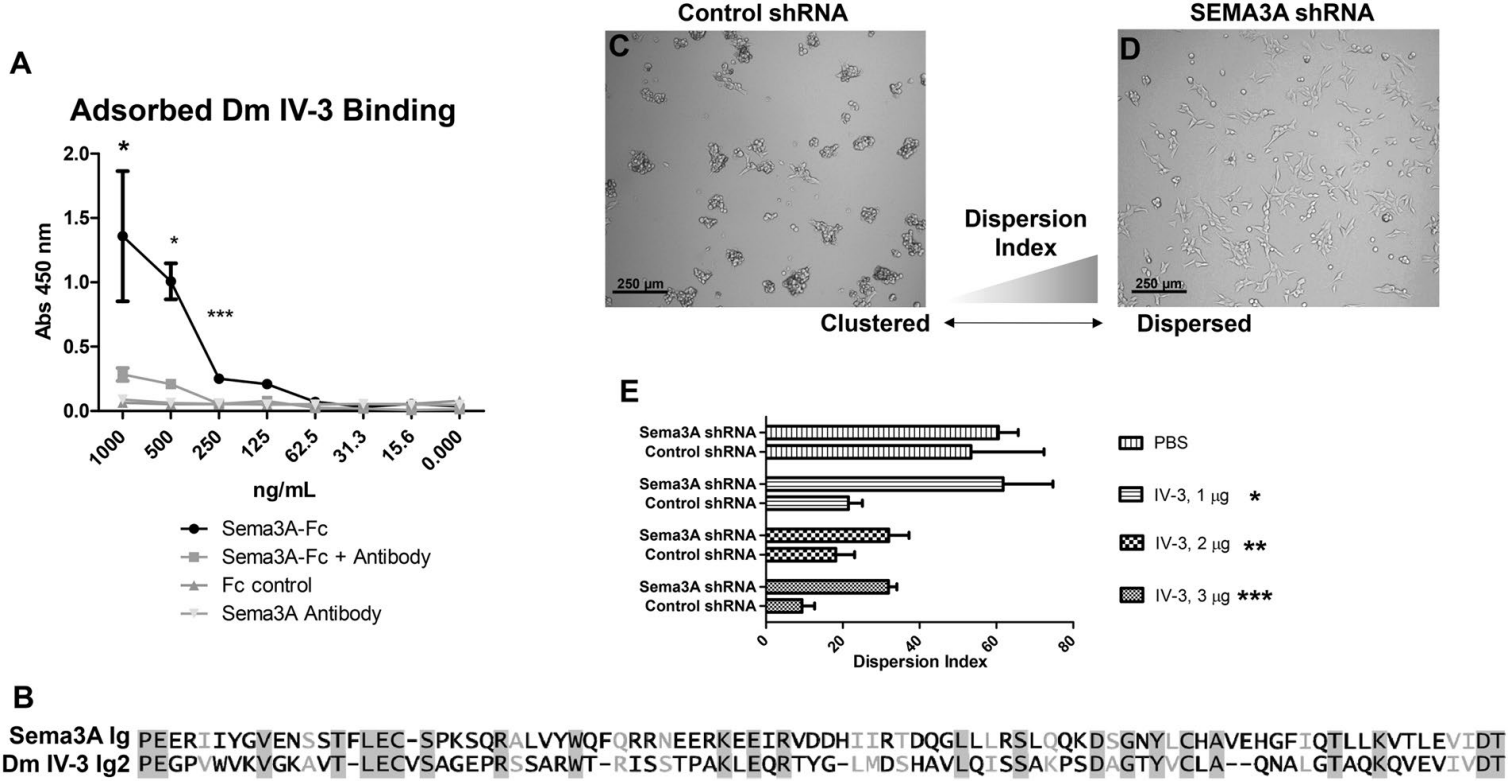
Sema3A binds Domain IV-3 and shRNA against Sema3A abrogates C4-2 cell clustering. (**A**) Domain IV-3 was adsorbed to well surfaces and subjected to a binding assay with Sema3A-Fc, Sema3A-Fc and Sema3A antibody, Sema3A antibody alone, and control IgG. Sema3A-Fc binds to Domain IV-3 and Sema3A antibody diminishes the binding signal significantly to 250 ng/mL (student’s unpaired t-test per concentration). (**B**) Blossom62 alignment of Ig 17 of perlecan (2^nd^ of Domain IV-3) and the Ig of Sema3A. C4-2 cells were seeded on Domain IV-3 after being transduced with either control shRNA (panel C) or Sema3A directed shRNA (panel D). (**E**) Dispersion index quantification between control and directed shRNA for each substrate type and amount. Significance values are student’s unpaired t-test between control and Sema3A shRNA. p-values: *<0.05, **<0.01, ***<0.001.

Because antibody engagement with Sema3A at the C-terminus induced clustering like that of Domain IV-3, we hypothesized that perlecan Domain IV-3 would bind to Sema3A. A binding assay with immobilized Domain IV-3 and Sema3A in solution indicates that the two extracellular proteins readily bind to one another (Fig. 6A). Domain IV-3 (1 µg/mL) was surface adsorbed, blocked, and incubated with various protein solutions, washed, and detected with protein A-HRP/TMB substrate. Controls, Sema3A antibody and non-immune IgG, showed no binding to Domain IV-3. In contrast, Sema3A-Fc bound Domain IV-3 over levels of internal controls (Sema3A-Fc incubated with immobilized BSA) over a range of concentrations extending to 125 ng/mL. The C-terminal directed Sema3A antibody successfully competed with Domain IV-3 to bind Sema3A-Fc and lower overall binding efficiency, which is statistically significant for the range of concentrations up to 250 ng/mL. This binding competition indicates Domain IV-3 and the antibody likely recognize similar protein regions in Sema3A. A comparison of the Ig domains of Domain IV-3 and Sema3A is depicted in the alignment in Fig. 6B. The second Ig of Domain IV-3 (17^th^ overall in full length perlecan) and the Ig domain of Sema3A have high sequence homology (26% identity, 39% positive, 6% gaps), opening the possibility for Ig-Ig homotypic-like interactions. QCM-D data corroborates the binding interaction between Domain IV-3 and Sema3A (supplemental Figure S2). Flowing Sema3A over the gold-sensor adsorbed with Domain IV-3 results in a frequency drop (time point 3) that persists during continued washing (time point 4). Flowing a control protein (BSA) over Domain IV-3 does not produce a similar frequency drop. Thus, Domain IV-3 and Sema3A can bind to each other, with *in silico* and experimental evidence pointing towards a homotypic like interaction through the Ig motifs of perlecan and the C-terminal Ig of Sema3A.

To next determine if C4-2 cells depend on Sema3A for perlecan Domain IV-3-induced clustering activity, cells were transduced either with scrambled control or *SEMA3A* transcript targeted shRNA viral vectors, then cultured either on control (PBS) or Domain IV-3 coated 96-well plates at three concentrations. Knockdown estimates by densitometric quantification of Western blot (Supplemental Figure S3) for this shRNA method indicate that knockdown of approximately 80% was achieved. Figure 6C and D present representative images of cells, either control (Fig. 6C) or *SEMA3A* shRNA targeted (Fig. 6D) grown on a Domain IV-3 substrate. Control shRNA had no effect on the ability of C4-2 cells to cluster on Domain IV-3. In contrast, *SEMA3A* targeted shRNA abrogated the ability of C4-2 cells to cluster on Domain IV-3, resulting in a higher dispersion index. These findings point to Sema3A as a key binding partner supporting perlecan Domain IV-3’s ability to induce clustering/repulsion effect in C4-2 PCa cells.

### MMP-7 Reverses Perlecan Domain IV-3 Clustering Effect

**Figure 7:**
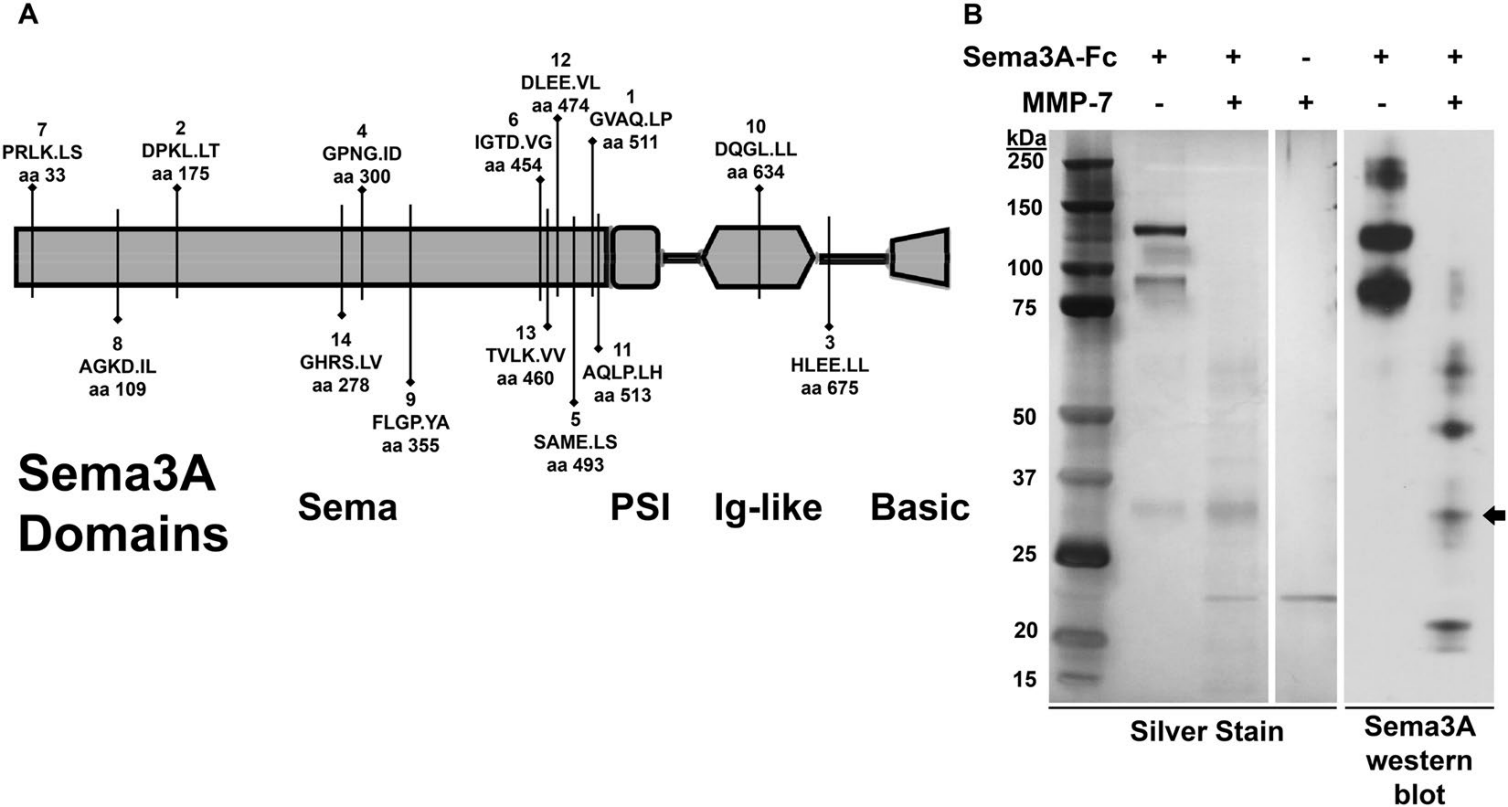
Recombinant Sema3A-Fc is digested by MMP-7. (**A**) MMP-7 *in silico* digestion of Sema3A using SitePrediction online software. Shown is a schematic of Sema3A with its domains (sema, plexin/semaphorin/integrin (PSI), immunoglobulin (Ig)-like, basic). Each line represents a cleavage site with > 95% specificity. The bolded number is the rank in average score. Below rank average score is the amino acid sequence with the P1 cleavage site (cleaves at period). Amino acid sequence is based on UniProt ID Q14563, which includes the signal sequence not shown in the schematic. (**B**) Sema3A-Fc (0.75 µg) was incubated alone or with MMP-7 (0.08 µg) overnight. A silver stain and western blot against Sema3A indicate MMP-7 fragments Sema3A into multiple smaller sub bands including a 30 kDa band indicated by an arrow.

The extracellular protease MMP-7 previously was shown to cleave perlecan in multiple contexts^9^. Cleavage of Sema3A by active MMP-7 in the metastatic microenvironment also could occur. To initially determine if Sema3A was a good substrate for MMP-7, an *in-silico* analysis was performed. A schematic of Sema3A and its domains is shown in Fig. 7A along with predicted cleavage sites demonstrating greater than 95% specificity. Each predicted site has an average score rank (1–14, 1 being highest probability to cleave) with the amino acid sequence and P1 site number (period indicates proteolytic site). The highest ranked site (amino acid 511) occurs just before the PSI domain, which would release a ~30 kDa C-terminal fragment. Additionally, MMP-7 is predicted to cleave the Ig domain itself. However, most cleavage sites are predicted to be at the end of the sema domain, potentially releasing the C-terminal portion including the Ig domain from a majority of the molecule. In Fig. 7B, recombinant Sema3A-Fc was incubated either alone or with MMP-7 overnight at room temperature. A silver stain and western blot for Sema3A (C-terminal directed rabbit antibody) demonstrated that MMP-7 successfully fragmented the larger bands into smaller sub bands. Notably, a new Sema3A positive band of approximately 30 kDa appears with MMP-7 incubation (indicated by the arrow), which likely is the C-terminal fragment predicted by the rank 1 cleavage site (Fig. 7A). Other bands barely sensitive enough to be detected by silver stain are detected by the Western blot. Unfortunately, Sema3A contains an Fc fragment complicating identification of the bands. Nevertheless, bands that are smaller than full length Sema3A are produced indicating it is cleaved by MMP-7. Taken together, these experiments demonstrate that Sema3A is likely to be cleaved by MMP-7/Matrilysin along with perlecan when both are present in the metastatic niche.

## Discussion

While we have come to understand perlecan’s role in forming tissue boundaries and how MMP-7 cleaves these boundaries to allow cancer cells to move through tissues^2,9,37–39^ knowledge of the cell surface components that interact with perlecan to influence PCa cell behavior long have remained elusive. Clues in the literature from studies of invertebrate development led us to look successfully for an interaction between human perlecan and a class of extracellular signaling molecules, the semaphorins. Specifically, we observed strong focal adhesion kinase (FAK) dephosphorylation by perlecan and its Ig rich region, Domain IV-3. In metastatic PCa, the semaphorin/plexin axis is not well studied, although there are hints of such an interaction in the literature. Multiple PCa cells express and release semaphorin 3A (Sema3A) to encourage osteoblastic differentiation of pre-osteoblastic MC3T3-E1 cells^40^. Class III semaphorins differentially affect PCa cells, in that Sema3A causes deadhesion and indolence and Sema3C overexpression produces more aggressive phenotypes^35^. Additionally, pro-oncogenic mutations in plexins have been reported in PCa tumors^41^. Overall, this study sought to mechanistically establish perlecan’s involvement in semaphorin signaling and to describe how MMP-7 effectively blocks this axis to favor dispersion and metastasis in PCa. Our work showed that full length perlecan initiates the same clustering as the 7 Ig-like repeat perlecan subdomain, Domain IV-3, and that both actions are reversed by MMP-7 activity. MMP-7 activity would not remove the GAG chains, thus this activity tracks to a new functional region of the core protein that is relatively near the previously described α_2_β_1_ integrin activating site in C-terminal Domain V^14,17,42^.

Modifications to FAK signaling revealed by the RPPA analysis meshed with the anti-adhesive effect observed in PCa cell culture. FAK is a common indicator of integrin engagement^43^ and FAK itself can modulate integrin activity and, by extension, adherence to ECM substrates^44^. Perlecan/Domain IV-3 antagonizes FAK activity by dephosphorylation at Y397. Modulation of FAK activity ostensibly links perlecan to the wide array of pro-tumorigenic FAK dependent processes including invasion, metastasis, survival, and growth^43,45–47^. In relation to FAK dephosphorylation, we observed a rapid clustering effect and immediate repulsion from the Domain IV-3 coated substrata. This effect is reminiscent of a previously unexplained phenomenon of repulsion/deadhesion to perlecan seen with hematopoietic cells^6^. We note that hematopoietic cells have Sema3 family proteins that are modulated in hematological cancers^48^. The authors found that the same preparation of full length perlecan was simultaneously pro-adhesive for fibroblasts, but anti-adhesive for several hematopoietic cells. Like the authors, we observed that increased FBS, which contains high amounts of pro-adhesive integrin binding fibronectin/vitronectin^49^, antagonize the clustering/repulsive effect of perlecan. Notably, repulsion of bone marrow derived cells was dependent on the protein core, and not the GAG chains^6^, just as we observed for multiple PCa cell lines. We thus propose that the 7 Ig-like repeat of Domain IV-3 contains the motifs responsible for repulsion of cell substratum adhesion in multiple cell types, favoring cell-cell adhesion.

Semaphorins are a class of extracellular signaling molecules that provide cell guidance cues by modifying adhesion and motility^25^. Several PCa cells express Sema3A and its receptor neuropilin-1 that in turn binds to multiple plexins^40,50^. Plexins have an intracellular portion with Ras-GAP (guanosine triphosphate (GTP)ase activation protein) activity. Sema3A initiates Ras-GAP activity within plexin-A1 to induce axon collapse or repulsion^51^. Semaphorin/plexin GAP functions can lead to integrin deactivation in human cells^52,53^. In *Drosophila* genetic-based studies, perlecan (aka trol) acted in concert with semaphorins and plexins to dephosphorylate FAK, deactivate integrins, and guide repulsive motor axons to defasciculate^18^. This guidance cue effect was dependent upon the protein core, and not the GAG chains present in domain I. Again, our data points to domain IV in *Drosophila* as the lead candidate within perlecan mediating the semaphorin interaction. The *Drosophila* domain IV comprises 10 Ig-like repeats^2^, suggesting that the Ig repeat structure may be needed to confer an activity requiring tandem presentation of active motifs.

**Figure 8:**
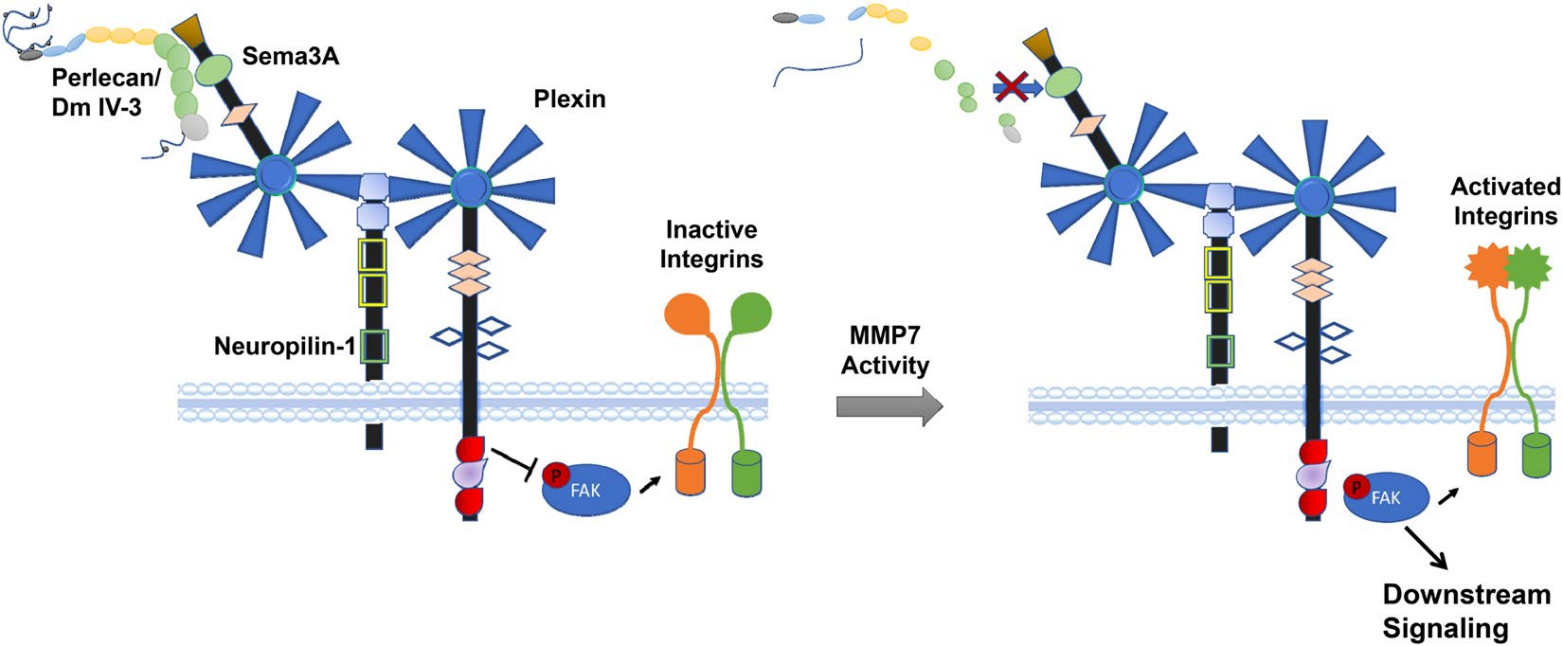
Model of perlecan/Sema3A proposed interactions. In non-invasive prostate overgrowth or early cancer (PCa), intact perlecan is present in the immediate stroma surrounding Sema3A expressing prostatic cells. In this state (left), perlecan engages Sema3A, stabilizing its interaction with the neuropilin-1 and plexin complex. Plexin Ras-GAP activity favors a state of focal adhesion kinase (FAK) dephosphorylation and integrin deactivation. In this state, PCa cell-cell adhesion dominates cell-substratum adhesion, abrogating tissue invasion. As PCa progresses, active MMPs such as MMP-7 can cleave perlecan and/or Sema3A. Ras-GAP activity of plexins is lost, allowing FAK phosphorylation to occur along with subsequent downstream signaling (right). FAK signaling reactivates integrins to favor substrata-mediated dispersion, as seen to occur during tumor dyscohesion and metastasis. GAP = guanosine triphosphate (GTP)ase activation protein.

Because Sema3A mitigates adhesion and motility in PCa cells, we tested for a physical interaction between Sema3A and perlecan Domain IV-3. An antibody that recognizes the C-terminus, including the Ig portion, of Sema3A when used as a substrate imitated Domain IV-3’s clustering effect on C4-2 cells, suggesting that Domain IV-3 and the antibody engage Sema3A at the same Ig module(s). Sema3A’s Ig module has high homology with Domain IV-3’s Ig repeats opening the possibility for Ig-Ig interactions similar to platelet endothelial cell adhesion molecule (PECAM) homotypic binding^54^. This idea is further supported by the finding that the same Sema3A antibody abrogates the binding between Sema3A and Domain IV-3. Sema3 proteins bind their neuropilin receptor via an N-terminal sema domain, leaving the C-terminus free to engage with other molecules, like perlecan^55–58^. Perlecan Domain IV-3 binding to Sema3A’s C-terminus may stabilize the Sema/neuropilin/Plexin complex to favor GAP plexin activity leading to FAK/integrin deactivation (model Fig. 8).

Our findings extend the understanding of the complex upstream extracellular matrix networks responsible for modifying FAK signaling. Downstream signaling of FAK is well studied in multiple cellular contexts including cancer^43,59^. This work links extracellular perlecan to intracellular FAK to influence major signaling networks aiding in cancer progression. For example, FAK coordinates signaling partners including ERK, PI3K/AKT, and Src to aid in invasion and metastasis^60^. In PCa, FAK and ERK work in concert to maintain attachment of PCa cells to substrata and ultimately to support an aggressive phenotype^47^. In other systems, FAK lies directly upstream of the PI3K/AKT pathway, acting as the mediator of β1 integrin attachment and signaling^61^. A new link, however, was revealed by the RPPA network between the transcription factor FoxM1 and FAK. Recently, FoxM1, acting in concert with another transcription factor, CENPF, was described as a master regulator of PCa metastasis^62^. How FAK could mechanistically affect FoxM1 is unknown, but both AKT and ERK are involved in FoxM1 signaling^63,64^. Regardless, finding perlecan can downregulate the pro-metastatic FoxM1 through semaphorin/FAK signaling could open exploration new therapeutic avenues for metastatic PCa.

Extracellular proteolytic enzymes, like MMP-7, are important modifiers of the tumor microenvironment to switch from a largely indolent pathology to active metastasis in multiple cancers including PCa^65–67^. Our work presents yet another avenue for MMP-7 to counteract innate anti-tumorigenic molecules. In prostate tissue, Sema3A and neuropilin-1 are expressed in glands and during the early carcinogenesis^68^. However, Sema3A glandular expression is decreased at later stages and in hormone refractory PCa^68^. Active MMP-7 was shown to be increased in PCa during metastatic progression^11^. These data, along with our observations that MMP-7 can cleave Sema3A, provide correlative evidence that MMP-7 may cleave Sema3A in the tumor microenvironment to aid metastasis. Potential cleavage sites identified *in silico*, point to MMP-7 releasing the C-terminal portion of Sema3A, which our data indicate is the binding site for perlecan. However, future experiments involving N-terminal sequencing and mass spectrometry are needed to biochemically verify MMP-7 generated Sema3A cleavage sites and fragments. Nevertheless, MMP-7 may generate a Sema3A “dummy” receptor for perlecan, removing its ability to promote cell-cell adhesion and limit dispersion.

The ability of perlecan and semaphorins to modulate androgen signaling and participate in castration resistant prostate cancer (CRPC) was not assessed directly in this study. However, perlecan has the ability to cluster isogenic cell lines that we studied representing every progression step: LNCaP (androgen sensitive), C4-2 (metastatic CRPC), and C4-2B (bone metastatic CRPC). In the transition from androgen sensitive to resistant, LNCaP and C4-2 cells continue to express similar levels of Sema3A^40^ allowing perlecan to engage with the sema/neuropilin/plexin axis. In PCa tissue, Sema3A protein decreases as seen by immunohistochemistry, but persists at the mRNA level^68^, pointing to proteolytic degradation in the extracellular environment. MMP-7 is the likely protease responsible given it readily degrades both Sema3A and perlecan^9^. The plexin-B1/Sema4D signaling axis is known to enhance androgen receptor (AR) signaling in low androgen environments likely to be encountered during androgen deprivation therapy^69^. Similar studies examining AR modification by Sema3A/neuropilin/plexin-A1 signaling have not been performed, but it generally opposes the plexin-B1/4D complex^70^. Therefore, destruction of Sema3A/perlecan signaling as we described may enhance Sema4D signaling to circumvent ADT, but future studies must be undertaken to test this idea.

We present a working model to test many future mechanistic studies, as seen in Fig. 8. In normal prostate or early PCa, perlecan is present in the immediate stroma and in the basal lamina surrounding Sema3A positive prostate glands. In this state, perlecan binds Sema3A in prostatic cells, stabilizing its interaction with neuropilin-1 and plexin. Plexins have Ras-GAP activity that stabilizes a state of FAK dephosphorylation and integrin deactivation, leading to PCa cell-cell adhesion being favored over cell-substratum adhesion. In this state, tissue invasion would not be expected. As PCa progresses, active MMPs, such as MMP-7, are increased, resulting in the potential proteolysis of both perlecan and Sema3A. Consequent to this, the Ras-GAP activity of plexins is lost, allowing FAK phosphorylation to occur along with subsequent downstream signaling including AKT and ERK. This activation leads to integrin reactivation to favor substrata-mediated dispersion and release from cell-cell adhesion, as seen to occur during tumor dyscohesion and metastasis. While many mechanistic details remain to be elucidated, the findings from this study lay out a novel border interaction between perlecan in the matrix and Sema3A in PCa cells that is disrupted by MMP-7 to favor an invasive FAK activated PCa phenotype.

## Electronic supplementary material

Supplementary Material

Video of perlecan domain IV induced clustering

## References

[1] Siegel RL, Miller KD, Jemal A (2016). Cancer statistics, 2016. CA. Cancer J. Clin..

[2] Farach-Carson MC, Warren CR, Harrington DA, Carson DD (2014). Border patrol: Insights into the unique role of perlecan/heparan sulfate proteoglycan 2 at cell and tissue borders. Matrix Biol..

[3] Rowe RG, Weiss SJ (2008). Breaching the basement membrane: who, when and how?. Trends Cell Biol..

[4] Warren CR, Grindel BJ, Francis L, Carson DD, Farach-Carson MC (2014). Transcriptional Activation by NFκB Increases Perlecan/HSPG2 Expression in the Desmoplastic Prostate Tumor Microenvironment. J. Cell. Biochem..

[5] Murdoch AD, Liu B, Schwarting R, Tuan RS, Iozzo RV (1994). Widespread expression of perlecan proteoglycan in basement membranes and extracellular matrices of human tissues as detected by a novel monoclonal antibody against domain III and by *in situ* hybridization. J. Histochem. Cytochem..

[6] Klein G (1995). Perlecan in human bone marrow: A growth-factor-presenting, but anti-adhesive, extracellular matrix component for hematopoietic cells. Matrix Biol..

[7] Whitelock JM, Melrose J, Iozzo RV (2008). Diverse cell signaling events modulated by perlecan. Biochemistry.

[8] Iozzo RV, Sanderson RD (2011). Proteoglycans in cancer biology, tumour microenvironment and angiogenesis. J. Cell. Mol. Med..

[9] Grindel BJJ (2014). Matrilysin/matrix metalloproteinase-7(MMP7) cleavage of perlecan/HSPG2 creates a molecular switch to alter prostate cancer cell behavior. Matrix Biol..

[10] Cardillo MR, Di Silverio F, Gentile V (2006). Quantitative immunohistochemical and *in situ* hybridization analysis of metalloproteinases in prostate cancer. Anticancer Res..

[11] Hashimoto K, Kihira Y, Matuo Y, Usui T (1998). Expression of matrix metalloproteinase-7 and tissue inhibitor of metalloproteinase-1 in human prostate. J. Urol..

[12] Powell WC (1993). Expression of the metalloproteinase matrilysin in DU-145 cells increases their invasive potential in severe combined immunodeficient mice. Cancer Res..

[13] Grindel B (2016). Perlecan/HSPG2 and matrilysin/MMP-7 as indices of tissue invasion: tissue localization and circulating perlecan fragments in a cohort of 288 radical prostatectomy patients. Oncotarget.

[14] Bix G (2004). Endorepellin causes endothelial cell disassembly of actin cytoskeleton and focal adhesions through alpha2beta1 integrin. J Cell Biol.

[15] Fuki IV, Iozzo RV, Williams K (2000). J. Perlecan heparan sulfate proteoglycan: a novel receptor that mediates a distinct pathway for ligand catabolism. J. Biol. Chem..

[16] Chakravarti S, Horchar T, Jefferson B, Laurie GW, Hassell JR (1995). Recombinant domain III of perlecan promotes cell attachment through its RGDS sequence. J Biol Chem.

[17] Bix G (2007). Endorepellin, the C-terminal angiostatic module of perlecan, enhances collagen-platelet responses via the alpha2beta1-integrin receptor. Blood.

[18] Cho JY, Chak K, Andreone BJ, Wooley JR, Kolodkin AL (2012). The extracellular matrix proteoglycan perlecan facilitates transmembrane semaphorin-mediated repulsive guidance. Genes Dev..

[19] Yoshida Y (2012). Semaphorin Signaling in Vertebrate Neural Circuit Assembly. Front. Mol. Neurosci..

[20] Takamatsu H, Kumanogoh A (2012). Diverse roles for semaphorin−plexin signaling in the immune system. Trends Immunol..

[21] Epstein JA, Aghajanian H, Singh MK (2015). Semaphorin Signaling in Cardiovascular Development. Cell Metab..

[22] Xu R (2014). Semaphorin 3A: A new player in bone remodeling. Cell Adh. Migr..

[23] Tamagnone L (2012). Emerging Role of Semaphorins as Major Regulatory Signals and Potential Therapeutic Targets in Cancer. Cancer Cell.

[24] Muratori, C. & Tamagnone, L. In *Advances in cancer research* **114**, 59–85 (2012). 10.1016/B978-0-12-386503-8.00003-X 22588056

[25] Capparuccia L, Tamagnone L (2009). Semaphorin signaling in cancer cells and in cells of the tumor microenvironment - two sides of a coin. J. Cell Sci..

[26] Drabkin H, Nasarre P, Gemmill R (2014). The emerging role of class-3 semaphorins and their neuropilin receptors in oncology. Onco. Targets. Ther..

[27] Morgado M (2016). Tumor necrosis factor-α and interferon-γ stimulate MUC16 (CA125) expression in breast, endometrial and ovarian cancers through NFκB. Oncotarget.

[28] Wijeratne, S. S. S. *et al*. Single molecule force measurements of perlecan/HSPG2: A key component of the osteocyte pericellular matrix. *Matrix Biol*. **50**, (2015). 10.1016/j.matbio.2015.11.001 PMC4808358 26546708

[29] Chiu Y-C (2016). Sustained delivery of recombinant human bone morphogenetic protein-2 from perlecan domain I - functionalized electrospun poly (ε-caprolactone) scaffolds for bone regeneration. J. Exp. Orthop..

[30] York H, Kornblau SM, Qutub AA (2012). Network analysis of reverse phase protein expression data: characterizing protein signatures in acute myeloid leukemia cytogenetic categories t(8;21) and inv(16). Proteomics.

[31] Grindel BJBJ, Rohe B, Safford SESE, Bennett JJJ, Farach-Carson MCMC (2011). Tumor necrosis factor-α treatment of HepG2 cells mobilizes a cytoplasmic pool of ERp57/1,25D_3_-MARRS to the nucleus. J. Cell. Biochem..

[32] Verspurten J, Gevaert K, Declercq W, Vandenabeele P (2009). SitePredicting the cleavage of proteinase substrates. Trends Biochem. Sci..

[33] Xie Y (2016). MMP7 interacts with ARF in nucleus to potentiate tumor microenvironments for prostate cancer progression *in vivo*. Oncotarget.

[34] Kitsukawa T (1997). Neuropilin-semaphorin III/D-mediated chemorepulsive signals play a crucial role in peripheral nerve projection in mice. Neuron.

[35] Herman JG, Meadows GG (2007). Increased class 3 semaphorin expression modulates the invasive and adhesive properties of prostate cancer cells. Int. J. Oncol..

[36] Rohm B, Ottemeyer A, Lohrum M, Püschel AW (2000). Plexin/neuropilin complexes mediate repulsion by the axonal guidance signal semaphorin 3A. Mech. Dev..

[37] Warren, C. R., Grindel, B. J., Francis, L., Carson, D. D. & Farach-Carson, M. C. Transcriptional activation by NFκB increases perlecan/HSPG2 expression in the desmoplastic prostate tumor microenvironment. *J*. *Cell*. *Biochem*. **115**, (2014). 10.1002/jcb.24788 PMC4091977 24700612

[38] Grindel, B. *et al*. Perlecan/HSPG2 and matrilysin/MMP-7 as indices of tissue invasion: Tissue localization and circulating perlecan fragments in a cohort of 288 radical prostatectomy patients. *Oncotarget* **7** (2016). 10.18632/oncotarget.7197 PMC4891130 26862737

[39] Tilakaratne WM (2009). Matrix metalloproteinase 7 and perlecan in oral epithelial dysplasia and carcinoma *in situ*: an aid for histopathologic recognition of their cell proliferation centers. J. Oral Pathol. Med..

[40] Liu F (2014). Prostate cancer cells induce osteoblastic differentiation via semaphorin 3A. Prostate.

[41] Wong OG-W (2007). Plexin-B1 mutations in prostate cancer. Proc. Natl. Acad. Sci. USA.

[42] Lord MS (2014). The role of vascular-derived perlecan in modulating cell adhesion, proliferation and growth factor signaling. Matrix Biol..

[43] Sulzmaier FJ, Jean C, Schlaepfer DD (2014). FAK in cancer: mechanistic findings and clinical applications. Nat. Rev. Cancer.

[44] Michael KE, Dumbauld DW, Burns KL, Hanks SK, García AJ (2009). Focal adhesion kinase modulates cell adhesion strengthening via integrin activation. Mol. Biol. Cell.

[45] Lu H (2014). KLF8 and FAK cooperatively enrich the active MMP14 on the cell surface required for the metastatic progression of breast cancer. Oncogene.

[46] Pylayeva Y (2009). Ras- and PI3K-dependent breast tumorigenesis in mice and humans requires focal adhesion kinase signaling. J. Clin. Invest..

[47] Johnson TR (2008). Focal Adhesion Kinase Controls Aggressive Phenotype of Androgen-Independent Prostate Cancer. Mol. Cancer Res..

[48] Wang Z (2005). KDR and Sema3 genes expression in bone marrow stromal cells and hematopoietic cells from leukemia patients and normal individuals. Hematology.

[49] Hayman EG, Pierschbacher MD, Suzuki S, Ruoslahti E (1985). Vitronectin—A major cell attachment-promoting protein in fetal bovine serum. Exp. Cell Res..

[50] Hu P (2011). Multiplexed quantum dot labeling of activated c-Met signaling in castration-resistant human prostate cancer. PLoS One.

[51] Rohm B, Rahim B, Kleiber B, Hovatta I, Püschel AW (2000). The semaphorin 3A receptor may directly regulate the activity of small GTPases. FEBS Lett..

[52] Barberis D (2004). Plexin signaling hampers integrin-based adhesion, leading to Rho-kinase independent cell rounding, and inhibiting lamellipodia extension and cell motility. FASEB J..

[53] Oinuma I, Katoh H, Negishi M (2006). Semaphorin 4D/Plexin-B1–mediated R-Ras GAP activity inhibits cell migration by regulating β_1_ integrin activity. J. Cell Biol..

[54] Paddock C, Zhou D, Lertkiatmongkol P, Newman PJ, Zhu J (2016). Structural basis for PECAM-1 homophilic binding. Blood.

[55] Janssen BJC (2010). Structural basis of semaphorin-plexin signalling. Nature.

[56] Antipenko A (2003). Structure of the Semaphorin-3A receptor binding module. Neuron.

[57] Takahashi T (1999). Plexin-neuropilin-1 complexes form functional semaphorin-3A receptors. Cell.

[58] Pascoe HG, Wang Y, Zhang X (2015). Structural mechanisms of plexin signaling. Progress in Biophysics and Molecular Biology.

[59] Lee BY, Timpson P, Horvath LG, Daly RJ (2015). FAK signaling in human cancer as a target for therapeutics. Pharmacology & therapeutics.

[60] Meng XN (2009). Characterisation of fibronectin-mediated FAK signalling pathways in lung cancer cell migration and invasion. Br. J. Cancer.

[61] Xia H, Nho RS, Kahm J, Kleidon J, Henke CA (2004). Focal adhesion kinase is upstream of phosphatidylinositol 3-kinase/Akt in regulating fibroblast survival in response to contraction of type I collagen matrices via a beta 1 integrin viability signaling pathway. J. Biol. Chem..

[62] Aytes A (2014). Cross-Species Regulatory Network Analysis Identifies a Synergistic Interaction between FOXM1 and CENPF that Drives Prostate Cancer Malignancy. Cancer Cell.

[63] Xie Y (2015). Induction of forkhead box M1 (FoxM1) by EGF through ERK signaling pathway promotes trophoblast cell invasion. Cell Tissue Res..

[64] Zhang C (2016). Gli1 promotes colorectal cancer metastasis in a Foxm1-dependent manner by activating EMT and PI3K-AKT signaling. Oncotarget.

[65] Ii M, Yamamoto H, Adachi Y, Maruyama Y, Shinomura Y (2006). Role of matrix metalloproteinase-7 (matrilysin) in human cancer invasion, apoptosis, growth, and angiogenesis. Exp. Biol. Med. (Maywood)..

[66] Chaudhary AK, Pandya S, Ghosh K, Nadkarni A (2013). Matrix metalloproteinase and its drug targets therapy in solid and hematological malignancies: An overview. Mutat. Res. Mutat. Res..

[67] Horejs C-M (2016). Basement membrane fragments in the context of the epithelial-to-mesenchymal transition. Eur. J. Cell Biol..

[68] Yacoub M (2009). Differential expression of the semaphorin 3A pathway in prostatic cancer. Histopathology.

[69] Williamson M, de Winter P, Masters JR (2016). Plexin-B1 signalling promotes androgen receptor translocation to the nucleus. Oncogene.

[70] Neufeld G, Kessler O (2008). The semaphorins: versatile regulators of tumour progression and tumour angiogenesis. Nat. Rev. Cancer.

