# Supplementary material for: Matrilysin/MMP-7 Cleavage of Perlecan/HSPG2 Complexed with Semaphorin 3A Supports FAK-Mediated Stromal Invasion by Prostate Cancer Cells
Source: Sci Rep. 2018 May 8;8:7262. doi: 10.1038/s41598-018-25435-3 (PMC5940808; doi:10.1038/s41598-018-25435-3)
Supplement: Supplementary file 1 — Supplementary Material [file 41598_2018_25435_MOESM1_ESM.pdf]

Title

Matrilysin/MMP-7 Cleavage of Perlecan/HSPG2 Complexed with Semaphorin 3A Supports FAK-Mediated Stromal Invasion by Prostate Cancer Cells

Authors

Brian J. Grindel[1,2+], Jerahme R. Martinez[1*], Tristen V. Tellman[2], Daniel A. Harrington[1,2], Hamim Zafar[3], Luay Nakhleh[3], Leland W. Chung[4], Mary C. Farach-Carson[1,2*]

Affiliations

[1]Department of BioSciences, Rice University, Houston, TX 77005, USA

[2]Department of Diagnostic and Biomedical Sciences, University of Texas Health Science Center at Houston, School of Dentistry, Houston, TX 77054, USA

[3]Department of Computer Science, Rice University, Houston, TX 77005, USA

[4]Uro-Oncology Research Program, Samuel Oschin Comprehensive Cancer Institute at Cedars-Sinai Medical Center, Los Angeles, CA 90048, USA

* To whom correspondence should be addressed:
Mary C. Farach-Carson
Department of Diagnostic and Biomedical Sciences
University of Texas Health Science Center at Houston, School of Dentistry
1941 East Road, BBS 4220
Houston TX 77054

Phone: 713-486-4438
E-mail: Mary.C.FarachCarson@uth.tmc.edu

[+]Present address: Department of Cancer Systems Imaging, Division of Diagnostic Imaging, The University of Texas MD Anderson Cancer Center, Houston, TX, 77030

[*]Present address: Department of Mechanical Engineering, University of Delaware, Newark, DE 19706, USA

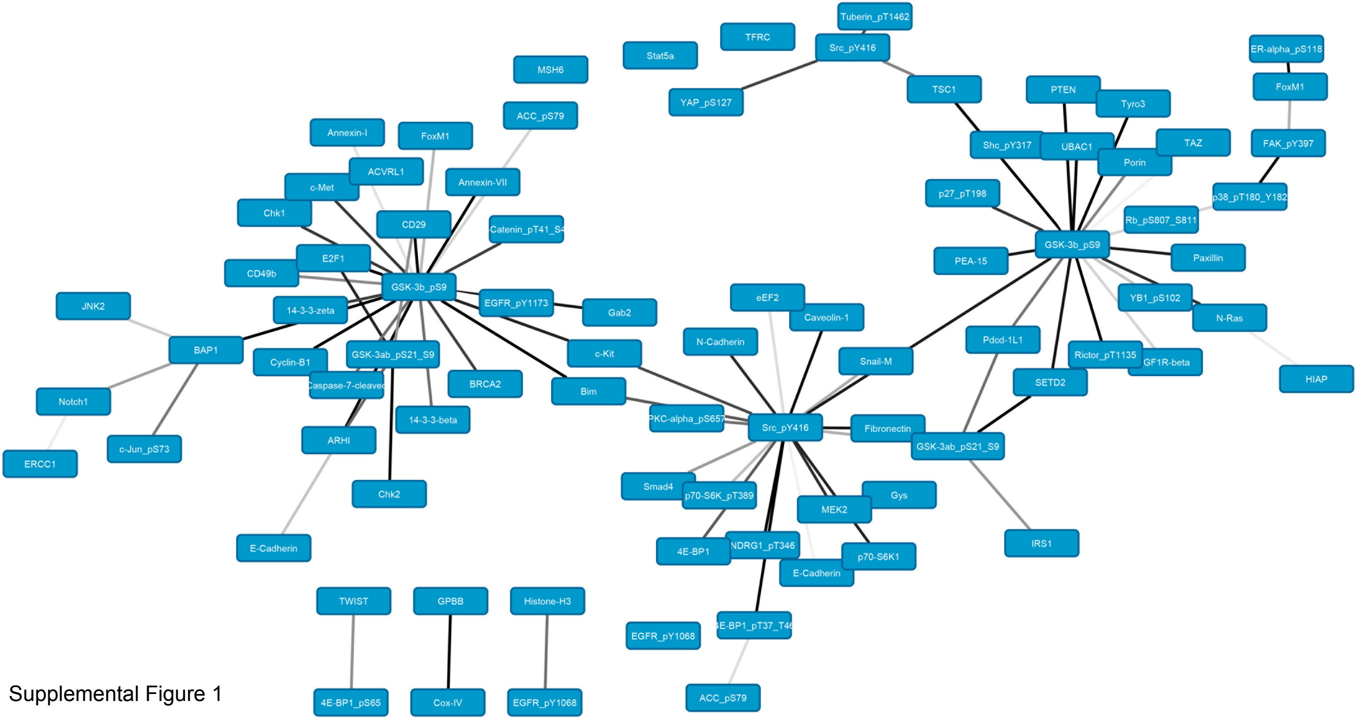

Supplemental Figure 1

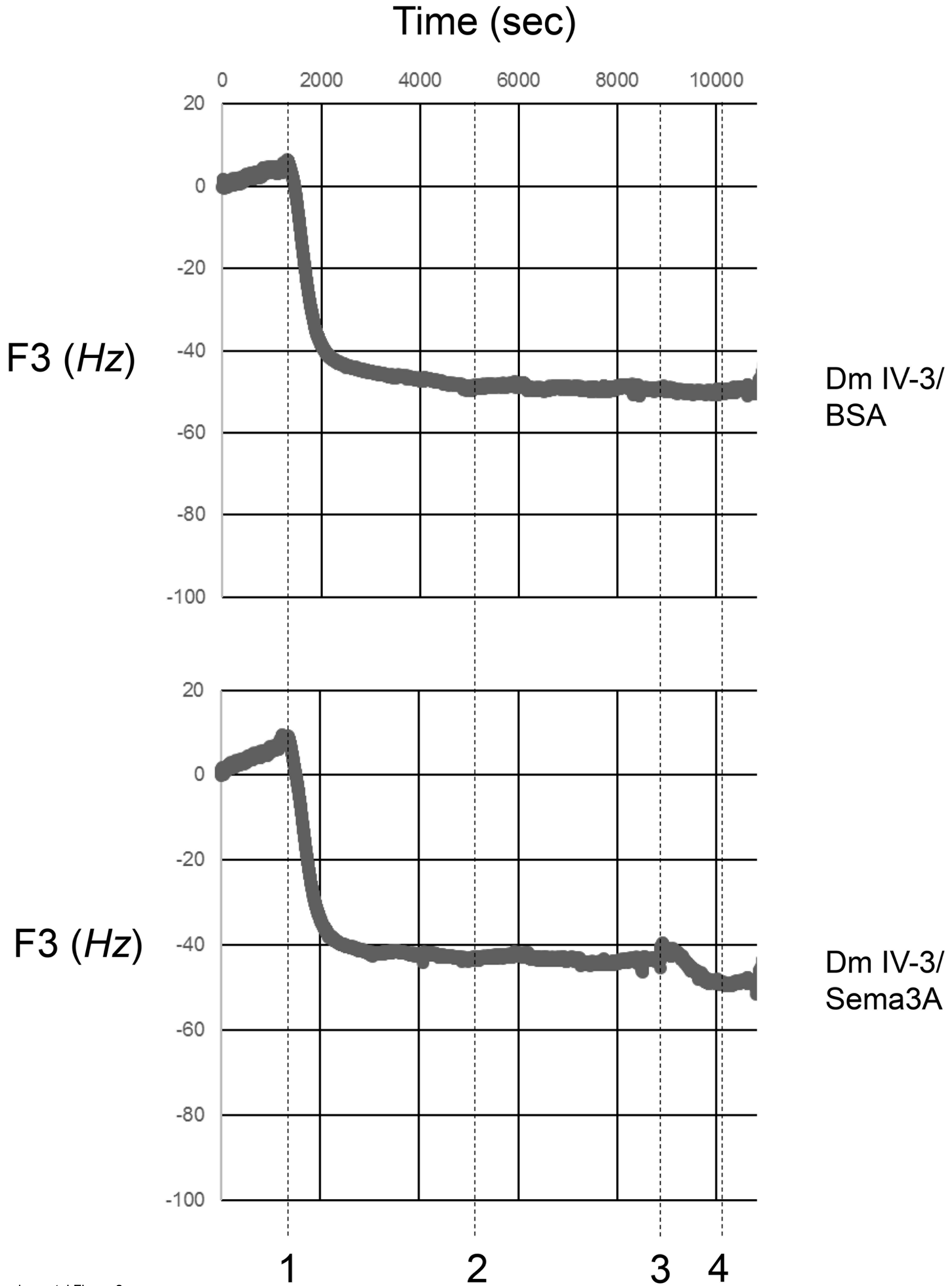

Supplemental Figure 2

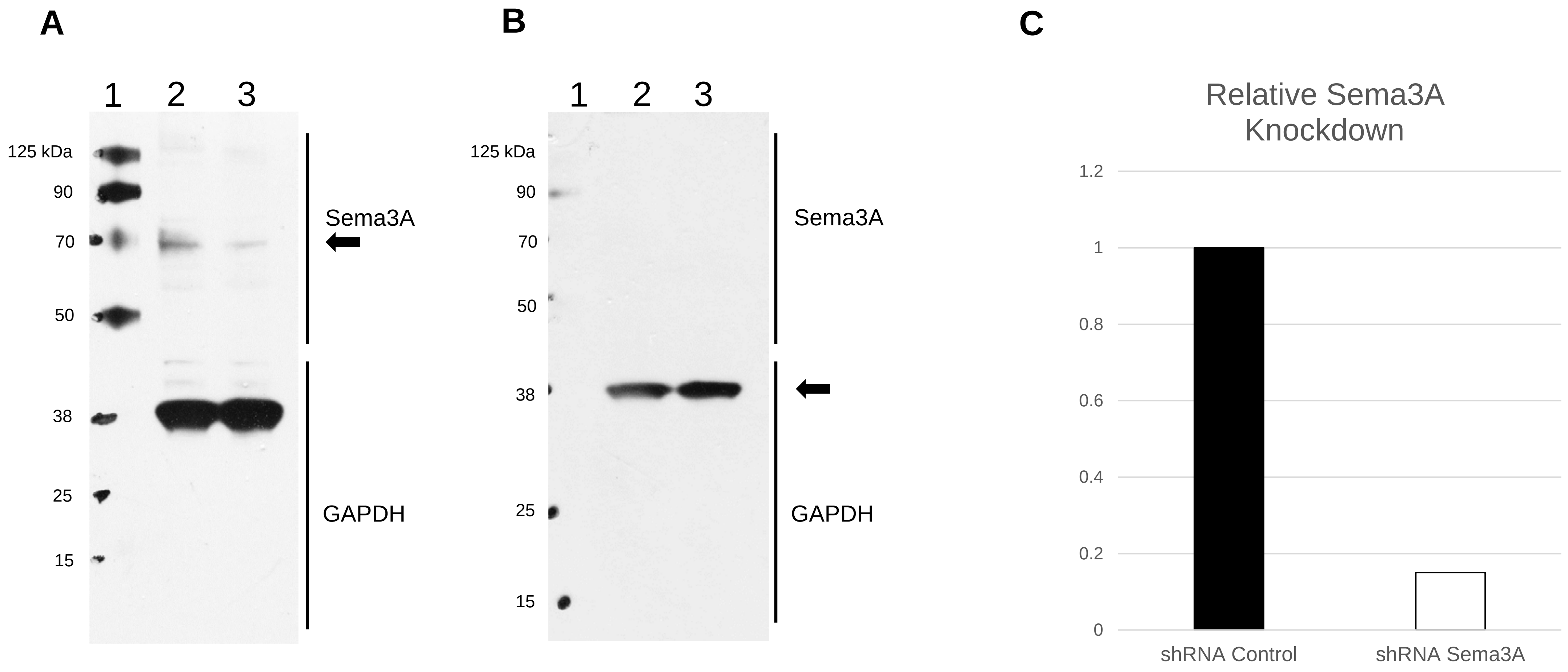

Supplemental Figure S3

Supplemental Figure S1: Network informed by the transition of C4-2 cells in their clustered state on intact Domain IV-3 to a dispersed state on MMP-7 cleaved Domain IV-3. Edges are created by iterative comparison of each protein-protein interaction between the two states through a student's T-test. RPPA results presented as a clustered heat diagram are provided in supplemental data file.

Supplemental Figure S2: Raw quartz crystal microbalance (QCM) data demonstrates binding interaction between perlecan Domain IV-3 (Domain IV-3) and Sema3A. Domain IV-3 was adsorbed to a gold sensor (time point 1), washed with binding buffer and then blocked in BSA (time point 2) and flowed with either control BSA or Sema3A (time point 3) and then washed with additional binding buffer (time point 4). Flowing Sema3A over Domain IV-3 (lower graph) results in a frequency drop that is retained during washing with the binding buffer. No frequency drop is observed with flowing BSA (upper graph).

Supplemental Figure S3: Sema3A protein levels are reduced by shRNA. C4-2 cells were treated for 24 hrs with either shRNA control or SEMA3A targeting shRNA . Whole cell lysates were probed for A) Sema3A and B) GAPDH. Lanes 1-3 show the molecular weight markers, shRNA control treated cell lysate, or shRNA sema3A treated cell lysates, respectively. C) Sema3A levels from shRNA control and shRNA treated cells were quantified using standard densitometry. A knockdown of near 80% was achieved.

Supplemental Video: Perlecan Domain IV-3 as a substrate induces rapid clustering of C4-2 cells. Live cell imaging (5% CO_2 atmosphere, 37°C) of C4-2 cells after being seeded into a cell culture well that has been spotted with 1.5 µg of Domain IV-3. Domain IV-3 is spotted approximately within the black fiduciary markers. Cells caught within the Domain IV-3 coated area immediately cluster while cells outside are unaffected. Tile imaged under 10X Objective and stitched together. Time lapse 4.5 hours with 10-minute intervals.

Supplemental data to support:

Title

Matrilysin/MMP-7 Cleavage of Perlecan/HSPG2 Complexed with Semaphorin 3A Supports FAK-Mediated Stromal Invasion by Prostate Cancer Cells

Authors

Brian J. Grindel[1,2+], Jerahme R. Martinez[1*] , Tristen V. Tellman[2], Daniel A. Harrington[1,2], Hamim Zafar[3], Luay Nakhleh[3], Leland W. Chung[4], Mary C. Farach-Carson[1,2*]

Affiliations

[1]Department of BioSciences, Rice University, Houston, TX 77005, USA

[2]Department of Diagnostic and Biomedical Sciences, University of Texas Health Science Center at Houston, School of Dentistry, Houston, TX 77054, USA

[3]Department of Computer Science, Rice University, Houston, TX 77005, USA

[4]Uro-Oncology Research Program, Samuel Oschin Comprehensive Cancer Institute at Cedars-Sinai Medical Center, Los Angeles, CA 90048, USA

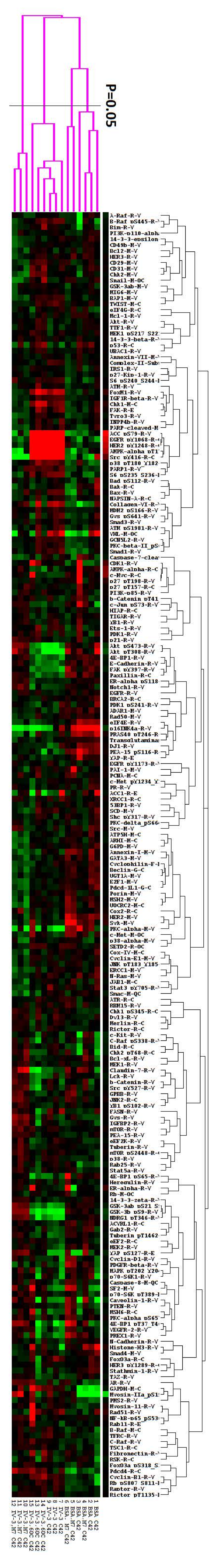
P=0.05
A-Raf-R-V
B-Raf_pS445-R-V
Bim-R-V
PI3K-p110-alpha
14-3-3-epsilon
CD49b-M-V
Bcl2-M-V
HER3-R-V
CD29-M-V
CD31-M-V
Chk2-M-V
Snail-M-QC
GSK-3ab-M-V
MIG6-M-V
BAP1-M-V
TWIST-M-C
eIF4G-R-C
Mcl-1-R-V
Akt-R-V
TTF1-R-V
MEK1_pS217_S221
14-3-3-beta-R-V
p53-R-C
UBAC1-R-V
Annexin-VII-M-V
Complex-II-Subunit
IRS1-R-V
p27-Kip-1-R-V
S6_pS240_S244-R
ATM-R-V
FoxM1-R-V
IGF1R-beta-R-V
Chk1-M-C
FAK-R-E
Tyro3-R-V
INPP4b-R-V
PARP-cleaved-M
ACC_pS79-R-V
EGFR_pY1068-R-C
HER2_pY1248-R-C
AMPK-alpha_pT172
Src_pY416-R-C
p38_pT180_Y182
PARP1-R-V
S6_pS235_S236-R
Bad_pS112-R-V
Bak-R-C
Bax-R-V
NAPSIN-A-R-C
Collagen-VI-R-V
MDM2_pS166-R-V
Gys_pS641-R-V
Smad3-R-V
ATM_pS1981-R-V
VHL-M-QC
GCN5L2-R-V
PKC-beta-II_pS660
Smad1-R-V
Caspase-7-cleaved
CDK1-R-V
AMPK-alpha-R-C
c-Myc-R-C
p27_pT198-R-V
p27_pT157-R-C
PI3K-p85-R-V
b-Catenin_pT41
c-Jun_pS73-R-V
HIAP-R-C
TIGAR-R-V
XB1-R-V
Ets-1-R-V
PDK1-R-V
p21-R-V
Akt_pS473-R-V
Akt_pT308-R-V
4E-BP1-R-V
E-Cadherin-R-V
FAK_pY397-R-V
Paxillin-R-C
ER-alpha_pS118
Notch1-R-V
EGFR-R-V
BRCA2-R-C
PDK1_pS241-R-V
ADAR1-M-V
Rad50-M-V
eIF4E-R-V
p16INK4a-R-V
PRAS40_pT246-R
Transglutaminase
DJ1-R-V
PEA-15_pS116-R
YAP-R-E
EGFR_pY1173-R
PAI-1-M-V
PCNA-M-C
c-Met_pY1234_Y1235
PR-R-V
ACC1-R-E
XRCC1-R-C
53BP1-R-V
SCD-M-V
Shc_pY317-R-V
PKC-delta_pS664
Src-M-V
ATP5H-M-C
ARHI-M-C
G6PD-M-V
Annexin-I-M-V
GATA3-M-V
Cyclophilin-F-M
Beclin-G-C
UGT1A-M-V
E2F1-M-V
Pdcd-1L1-G-C
Porin-M-V
MSH2-M-V
UQCRC2-M-C
Cox2-R-C
HER2-M-V
Syk-M-V
PKC-alpha-M-V
c-Met-M-QC
p38-alpha-M-V
SETD2-R-QC
Cox-IV-R-C
Cyclin-E1-M-V
JNK_pT183_Y185
ERCC1-M-V
N-Ras-M-V
JAB1-M-C
Stat3_pY705-R
Smac-M-QC
ATR-R-C
RBM15-R-V
Chk1_pS345-R-C
Dvl3-R-V
Merlin-R-C
Rictor-R-C
c-Kit-R-V
C-Raf_pS338-R
Bid-R-C
Chk2_pT68-R-C
Bcl-xL-R-V
MEK1-R-V
Claudin-7-R-V
Lck-R-V
b-Catenin-R-V
Src_pY527-R-V
GPBB-R-V
JNK2-R-C
YB1_pS102-R-V
FASN-R-V
Gys-R-V
IGFBP2-R-V
mTOR-R-V
PEA-15-R-V
eEF2K-R-V
Tuberin-R-V
mTOR_pS2448-R
p38-R-V
Rab25-R-V
Stat5a-R-V
4E-BP1_pS65-R
Heregulin-R-V
ER-alpha-R-V
Rb-M-QC
14-3-3-zeta-R
GSK-3ab_pS21_S9
GSK-3b_pS9-R-V
NDRG1_pT346-R-V
ACVRL1-R-C
Gab2-R-V
Tuberin_pT1462
eEF2-R-C
MEK2-R-V
YAP_pS127-R-E
Cyclin-D1-R-V
PDGFR-beta-R-V
MAPK_pT202_Y204
p70-S6K1-R-V
Caspase-8-M-QC
SF2-M-V
p70-S6K_pT389-R
Caveolin-1-R-V
PTEN-R-V
MSH6-R-C
PKC-alpha_pS657
4E-BP1_pT37_T46
VEGFR-2-R-V
PREX1-R-V
N-Cadherin-R-V
Histone-H3-R-V
Smad4-M-V
Foxo3a-R-C
HER3_pY1289-R
Stathmin-1-R-V
TAZ-R-V
AR-R-V
GAPDH-M-C
Myosin-IIa_pS1943
PMS2-R-V
Myosin-11-R-V
Rad51-R-V
NF-kB-p65_pS536
Rab11-R-E
B-Raf-M-C
TFRC-R-V
C-Raf-R-V
TSC1-R-C
Fibronectin-R-C
RSK-R-C
FoxO3a_pS318_S321
Pdcd4-R-C
Cyclin-B1-R-V
Rb_pS807_S811-R
Raptor-R-V
Rictor_pT1135-R

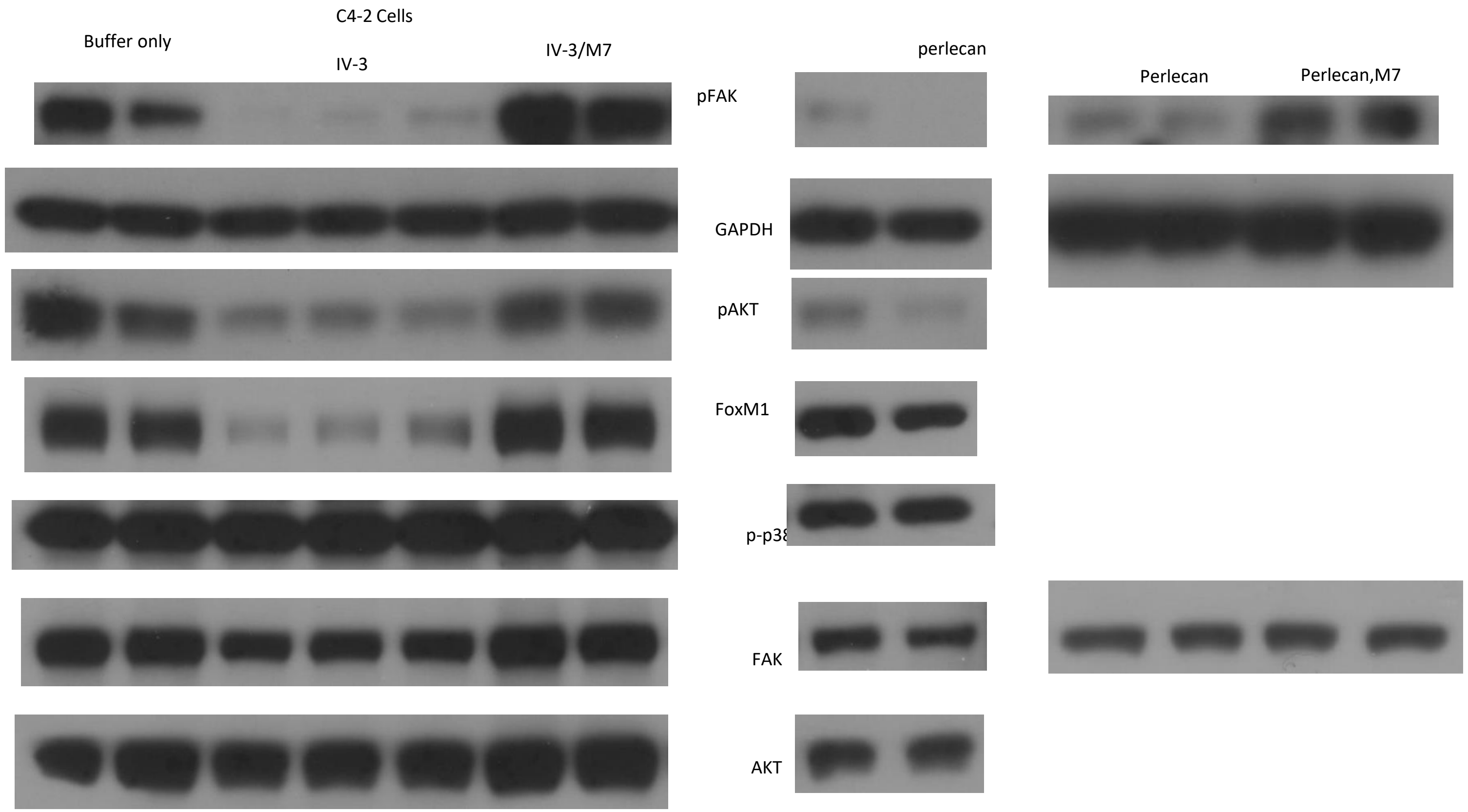

C4-2 Cells
Buffer only
IV-3
IV-3/M7
perlecan
Perlecan
Perlecan,M7
pFAK
GAPDH
pAKT
FoxM1
p-p38
FAK
AKT

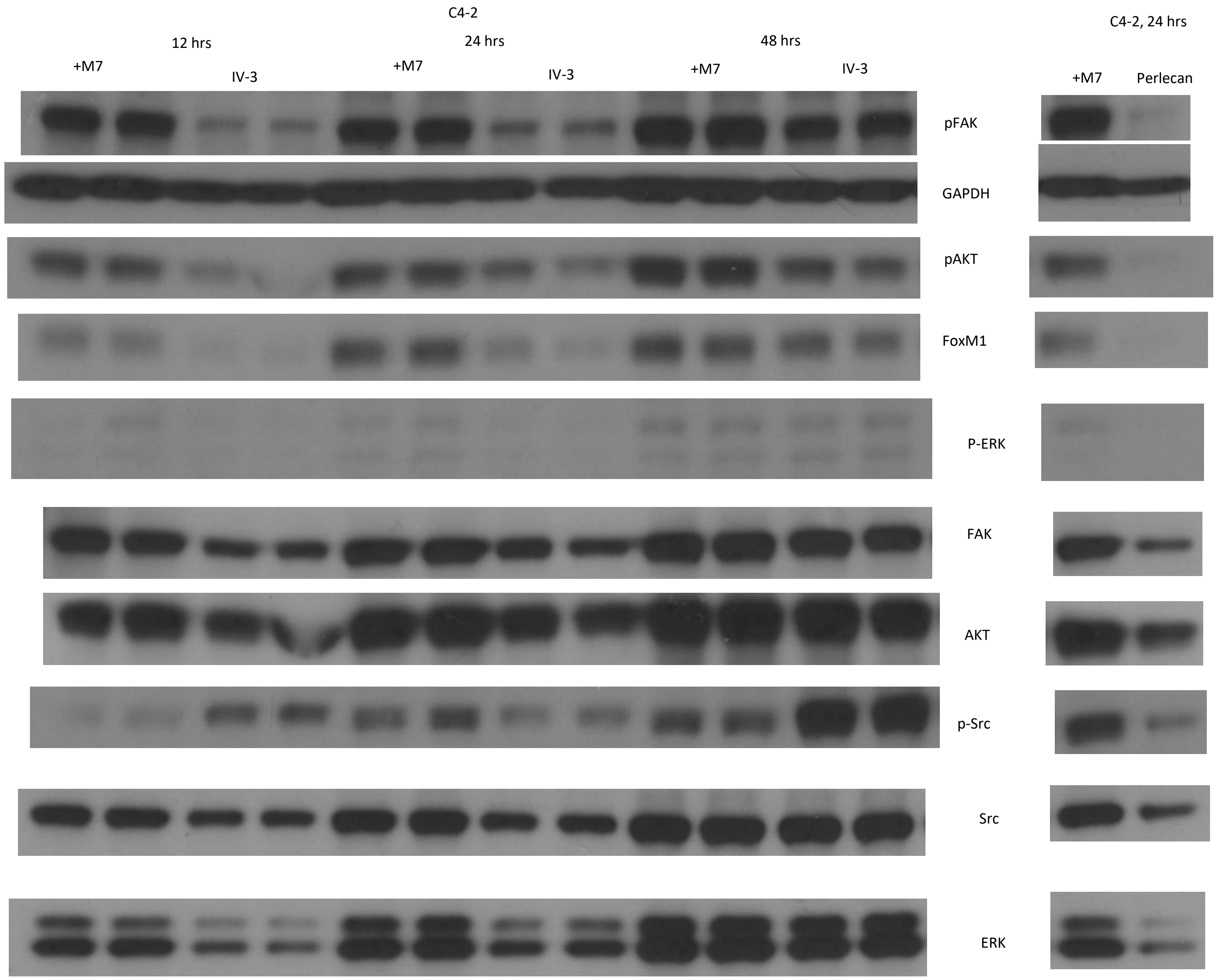
C4-2
12 hrs
24 hrs
48 hrs
+M7
IV-3
+M7
IV-3
+M7
IV-3
C4-2, 24 hrs
+M7
Perlecan
pFAK
GAPDH
pAKT
FoxM1
P-ERK
FAK
AKT
p-Src
Src
ERK

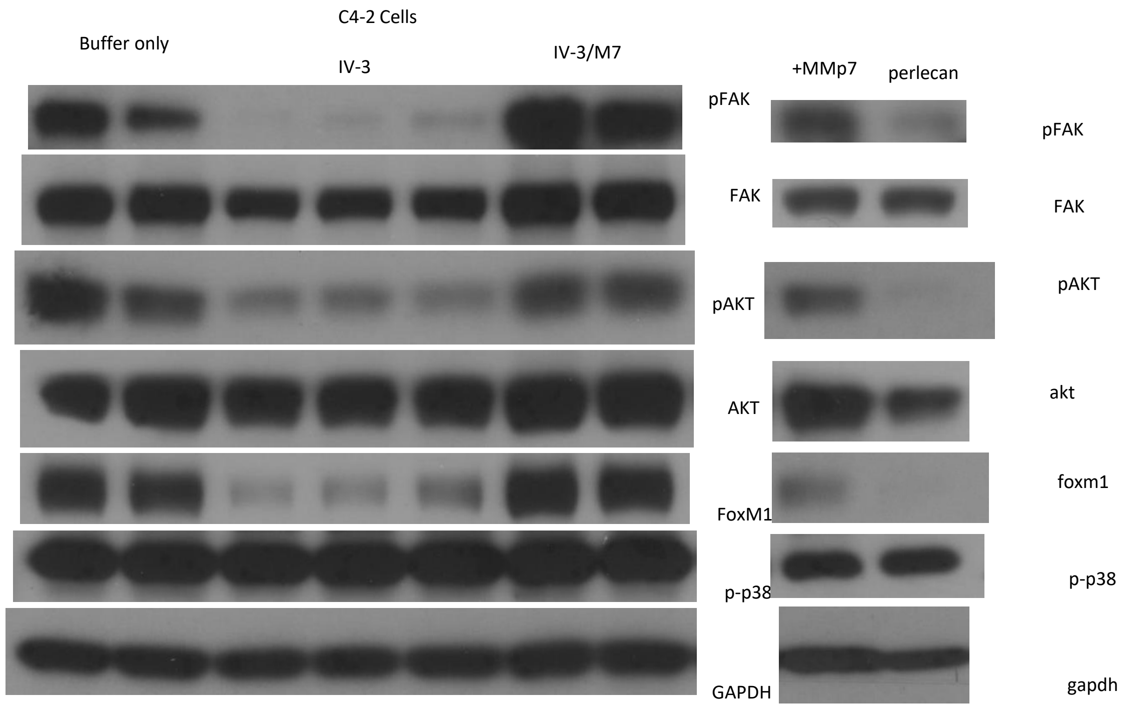
C4-2 Cells
Buffer only
IV-3
IV-3/M7
+MMp7
perlecan
pFAK
FAK
pAKT
AKT
FoxM1
p-p38
GAPDH
pFAK
FAK
pAKT
akt
foxm1
p-p38
gapdh

C42, 24 hrs

IV-3 Rabbit IgG Sema3aAb BSA

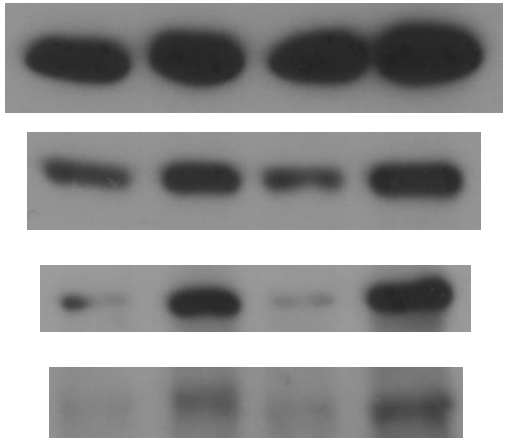

GAPDH

pAKT

FoxM1

pFAK

Rabbit IgG Sema3a Ab

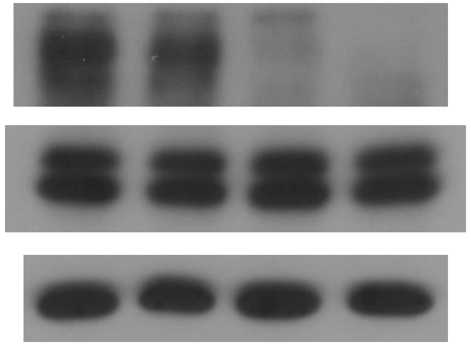

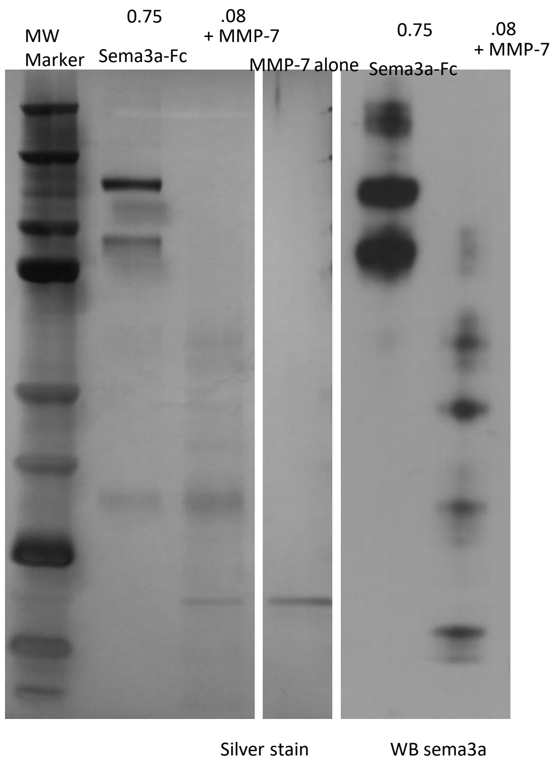
MW
Marker
0.75
Sema3a-Fc
.08
+ MMP-7
MMP-7 alone
0.75
Sema3a-Fc
.08
+ MMP-7
Silver stain
WB sema3a
